# Rabbit Litter-Derived Carbon Materials for Organophosphate Pesticide Mitigation: Adsorption Performance, Neurotoxicity Reduction, and Genotoxicity Assessment

**DOI:** 10.3390/jox16030075

**Published:** 2026-04-29

**Authors:** Tamara Lazarević-Pašti, Tamara Terzić, Andreja Leskovac, Sandra Petrović, Vedran Milanković, Nevena Radivojević, Jugoslav Krstić, Katarina Kokanov Stanković, Ana Jocic, Snežana Brković, Igor Pašti

**Affiliations:** 1 VINČA Institute of Nuclear Sciences—National Institute of the Republic of Serbia, University of Belgrade, Mike Petrovica Alasa 12–14, 11000 Belgrade, Serbia; tamara.tasic@vin.bg.ac.rs (T.T.); andreja@vin.bg.ac.rs (A.L.); sandra@vin.bg.ac.rs (S.P.); vedran.milankovic@vin.bg.ac.rs (V.M.); nevena.arsenijevic@vin.bg.ac.rs (N.R.); ana.jocic@vin.bg.ac.rs (A.J.); snezana.miulovic@vin.bg.ac.rs (S.B.); 2 Institute of Chemistry, Technology and Metallurgy, National Institute of the Republic of Serbia, University of Belgrade, Njegoševa 12, 11000 Belgrade, Serbia; jugoslav.krstic@ihtm.bg.ac.rs; 3 Faculty of Physical Chemistry, University of Belgrade, Studentski Trg 12–16, 11158 Belgrade, Serbia; 20240319@student.ffh.bg.ac.rs (K.K.S.); igor@ffh.bg.ac.rs (I.P.); 4 Serbian Academy of Sciences and Arts, Kneza Mihaila 35, 11000 Belgrade, Serbia

**Keywords:** chlorpyrifos, malathion, dimethoate, remediation, acetylcholinesterase inhibition, waste-derived carbon

## Abstract

Organophosphate pesticides are widely used agricultural chemicals that pose significant environmental and health risks due to their neurotoxicity, which is associated with inhibition of acetylcholinesterase. In this study, carbon materials derived from rabbit litter-based precursors were investigated as sustainable adsorbents for the removal of organophosphate pesticides from aqueous systems. The prepared materials exhibited a broad range of textural properties, with specific surface areas ranging from 10 to 487 m^2^ g^−1^, depending on the precursor composition. Adsorption experiments demonstrated measurable removal of chlorpyrifos, malathion, and dimethoate, with maximum adsorption capacities reaching 71.8 mg g^−1^ for malathion, although adsorption performance varied among materials, indicating a combined influence of pore accessibility and surface chemical heterogeneity. Evaluation of acetylcholinesterase inhibition before and after adsorption showed a consistent decrease in enzyme inhibition across all systems, with values reduced from 40% to as low as 20% for chlorpyrifos, from 35% to as low as 11% for malathion, and from 20% to as low as 10% for dimethoate, indicating a reduction in the neurotoxic potential of the treated solutions. In addition, the genotoxicity of the carbon materials varied with their structural and compositional characteristics, underscoring the importance of considering both adsorption performance and biological interactions. These findings demonstrate that waste-derived carbon materials can contribute to the removal of organophosphate contaminants while simultaneously reducing their associated neurotoxic effects.

## 1. Introduction

Organophosphate pesticides (OPs) are a major class of agricultural chemicals widely used to control insect pests in crop production [1]. Due to their extensive application and frequent release into surface waters and soils through agricultural runoff, these compounds are increasingly recognized as environmentally relevant xenobiotics [2]. Among the most commonly detected OPs are chlorpyrifos (CHP), malathion (MLT), and dimethoate (DMT), which are regularly reported in agricultural drainage systems and contaminated water bodies [3,4]. The use and environmental occurrence of OPs are regulated through established guidelines and maximum residue limits, reflecting their recognized impact on environmental and human health [5,6]. The environmental presence of these compounds raises significant toxicological concerns because organophosphates exert their biological effects primarily by inhibiting acetylcholinesterase (AChE), leading to disruption of cholinergic neurotransmission and causing neurotoxic outcomes in exposed organisms [7]. Mitigation of OP contamination, therefore, represents an important environmental and public health challenge. Among available treatment strategies, adsorption-based approaches are widely regarded as one of the most effective and operationally simple methods for removing organic contaminants from aqueous environments, particularly at relatively low concentrations [8]. Carbon-based materials are especially attractive adsorbents due to their high surface area, tunable porosity, chemical stability, and broad adsorption affinity toward hydrophobic and moderately polar organic molecules [9]. Conventional activated carbons, however, are often produced from non-renewable biomass precursors through energy-intensive processes, which limits their sustainability and increases production costs [10].

In recent years, increasing attention has been directed toward the development of carbon adsorbents derived from waste biomass and agricultural by-products [11,12,13]. Such materials offer a promising route to integrate environmental remediation with circular-economy principles by converting waste streams into functional materials [14]. A wide range of lignocellulosic residues has been explored as carbon precursors [15,16,17], yet livestock-associated wastes remain comparatively underinvestigated despite their continuous generation and complex chemical composition. Rabbit litter represents a particularly interesting example of such waste streams, as it typically contains a heterogeneous mixture of bedding material, urine residues, and droppings. The coexistence of lignocellulosic components, mineral phases, and nitrogen-containing compounds may result in carbon materials with distinct textural and chemical properties after pyrolysis.

While numerous studies have investigated the adsorption efficiency of carbon materials toward pesticides [18,19,20], significantly fewer studies have addressed the biological relevance of contaminant removal. In the context of xenobiotic mitigation, it is important not only to evaluate the capacity of materials to remove contaminants from solution, but also to assess whether such removal effectively reduces their biological activity [21,22,23]. For OPs, AChE inhibition is a well-established biomarker of neurotoxic potential and a relevant functional indicator of detoxification [24,25]. In addition to evaluating the biological effects of the contaminants themselves, the potential biological interaction of newly developed carbon materials should also be considered [26,27]. Fine carbon particles may come into contact with biological systems during synthesis, handling, or unintended environmental release [28,29,30]. Consequently, preliminary biological evaluation of such materials may provide valuable insight into their potential cellular interactions and safety profiles.

In this context, the present study investigates carbon materials derived from various rabbit litter precursors as sustainable adsorbents for the removal of OPs from aqueous systems. The materials were characterized for morphology, composition, and textural properties, and their adsorption behavior toward CHP, MLT, and DMT was examined. The biological relevance of pesticide removal was further evaluated by measuring AChE inhibition, while the genotoxicity of the materials was assessed using the cytokinesis-block micronucleus assay. The main objective of this study is to elucidate how pore structure, surface accessibility, and compositional heterogeneity influence both adsorption performance and biologically relevant outcomes, including neurotoxicity reduction and cytogenetic response. By integrating physicochemical characterization, adsorption analysis, and biological response assessment, this study provides a broader evaluation of waste-derived carbon materials for mitigating environmentally relevant xenobiotics.

## 2. Materials and Methods

### 2.1. Material Synthesis and Characterization

Not used rabbit litter (NRL), spent rabbit litter urine (SRL (U)), rabbit droppings (RD), and spent rabbit litter urine and rabbit droppings (SRL (U,RD)) were used as precursors. The precursor materials are heterogeneous biomass mixtures without a defined particle-size distribution. Pyrolysis was performed in a tube furnace (Protherm Electrical Tube Furnace, Protherm Furnaces, Ankara, Turkey) under a nitrogen atmosphere at a 100 L h^−1^ flow rate. The samples were heated to 650 °C at 5 °C min^−1^ and maintained at that temperature for 60 min before being cooled to room temperature under a nitrogen atmosphere. After pyrolysis, the resulting carbon materials consisted of irregular particles.

To remove soluble impurities and mineral residues, 100 mg of the obtained carbon materials was weighed and sequentially washed with 50 mL of 0.1 M sodium hydroxide (NaOH) (Centrochem, Stara Pazova, Serbia), followed by 50 mL of 0.1 M hydrochloric acid (HCl) (Centrochem, Stara Pazova, Serbia), and 50 mL of deionized water. The NaOH/HCl treatment was primarily applied to remove residual inorganic components and soluble impurities from the carbonized materials. Although minor changes in surface chemistry cannot be excluded, this step was not intended as a surface modification procedure, but rather to ensure consistent and comparable adsorption behavior. It should be noted that the reagent consumption in this step may be optimized in future applications, particularly in the context of scale-up and process sustainability. Finally, material was suspended in 50 mL of 50% ethanol (J.T. Baker, Phillipsburg, NJ, USA) to obtain a stock suspension at 2 mg mL^−1^. Final concentration of adsorbents in all experiments was 1 mg mL^−1^. The adsorbent concentration was selected as a representative dosage commonly used in batch adsorption studies, providing measurable removal while enabling comparison of adsorption performance across different materials.

The morphology and elemental composition of the samples were analyzed using a PhenomProX scanning electron microscope (SEM) (Thermo Fisher Scientific, Waltham, MA, USA) equipped with energy-dispersive X-ray spectroscopy (EDX).

Nitrogen adsorption–desorption measurements were performed at 77 K using a Sorptomatic 1990 apparatus (Thermo Finnigan, Parma, Italy). Before the analysis, the samples were degassed at 200 °C for 24 h. The obtained N_2_ isotherms were analyzed using ADP 5.13 software (Thermo Electron, Waltham, MA, USA). The specific surface area (*S*_BET_) was calculated using the Brunauer–Emmett–Teller (BET) equation [31], applied to the portion of the adsorption isotherm selected according to the Rouquerol criteria [32,33]. The total pore volume (*V*_tot_) was estimated by applying the Gurvich rule [31] at a relative pressure (*p*/*p*_0_) of 0.98. The micropore volume (*V*_mic_) and median micropore diameter (*d*_mic_) were determined using the Horvath-Kawazoe (HK) method [34], while the median mesopore diameter (*d*_mes_) was obtained using the Barrett-Joyner-Halenda (BJH) model [35] from the desorption branch of the isotherm.

A Fourier-transform infrared (FTIR) spectra were obtained using a Nicolet iS20 FT-IR spectrophotometer (Thermo Fisher Scientific, Waltham, MA, USA) equipped with an attenuated total reflectance (ATR) accessory. The spectral data were acquired over the wavenumber range 4000–400 cm^−1^, with each measurement comprising 64 scans at a resolution of 4 cm^−1^.

### 2.2. Adsorption Experiments

Batch adsorption experiments were performed to investigate the removal of OPs using the obtained carbon materials. The desired concentrations of both the adsorbent (1 mg mL^−1^) and the OPs DMT, MLT, and CHP (Pestanal, Sigma-Aldrich, Søborg, Denmark) were obtained by adding the appropriate amounts of stock solutions. The adsorption mixtures were incubated at 25 °C for specified time intervals on an Orbital Shaker-Incubator ES-20 (Grant-Bio, Cambridgeshire, UK) to allow sufficient adsorption. After incubation, the samples were centrifuged at 14,500× *g* for 10 min, followed by filtration through a 220 nm nylon syringe filter (KX Syringe Filter, Kinesis, Cole Parmer, St. Neots, UK). The concentration of OPs in the filtrate was determined by ultra-performance liquid chromatography (UPLC). The adsorption efficiency was calculated using the formula given in the Supplementary Materials. To ensure that no degradation occurred during the experiments, control samples without the carbon adsorbent were analyzed.

DMT, MLT, and CHP were quantified using a Waters ACQUITY UPLC system equipped with a photodiode array (PDA) detector and controlled by Empower software version 3. Separation was performed on an ACQUITY UPLC™ BEH C18 column (1.7 μm, 100 mm × 2.1 mm, Waters GmbH, Eschborn, Germany) under isocratic conditions. The mobile phase consisted of 10% acetonitrile in water (*v*/*v*) as phase A and pure acetonitrile as phase B. For DMT analysis, the mobile phase composition was 80% A and 20% B. For MLT, the composition was 40% A and 60% B, whereas for CHP it was 20% A and 80% B. The flow rate was 0.2 mL min^−1^, and the injection volume was 5 μL. Detection was performed at 200 nm for all OPs.

The adsorption kinetics of OPs onto the prepared carbon materials were evaluated using nonlinear kinetic models, including pseudo-first-order (PFO), pseudo-second-order (PSO), Elovich, and intraparticle diffusion models. Equilibrium adsorption behavior was analyzed using nonlinear isotherm models, including the Freundlich, Langmuir, Temkin, Dubinin-Radushkevich, and Sips models. The corresponding equations used for kinetic and isotherm modeling are provided in the Supplementary Materials.

Kinetic experiments were performed at an initial OP concentration of 1 × 10^−5^ mol dm^−3^ for all investigated pesticides. Adsorption isotherms were obtained using different initial concentration ranges depending on the pesticide: 5 × 10^−6^–5 × 10^−4^ mol dm^−3^ for CHP and MLT, and 5 × 10^−7^–5 × 10^−3^ mol dm^−3^ for DMT. The concentration ranges for adsorption isotherms were selected based on the solubility of the pesticides and the analytical detection limits, in order to ensure adequate coverage of the isotherm profiles for each compound. The initial pH values of the suspensions containing the investigated carbon materials were measured before the adsorption experiments and were 7.89 for NRL, 7.85 for RDs, 7.77 for SRL(U), and 7.07 for SRL(U,RDs).

### 2.3. AChE Inhibition Assay

The neurotoxic potential of OP solutions, both before and after adsorption, was evaluated by measuring AChE inhibition using a modified Ellman method [36]. In the assay, 1 IU of commercially purified AChE from electric eel (Sigma-Aldrich, Taufkirchen, Germany) was incubated with OP solutions (final concentration 10^−5^ mol dm^−3^) in 50 mM phosphate buffer (pH 8.0) at 37 °C for 20 min. The total reaction volume was 0.650 mL. The enzymatic reaction was initiated by the addition of acetylcholine iodide (AChI, Sigma-Aldrich, Taufkirchen, Germany) as substrate together with 5,5′-dithiobis(2-nitrobenzoic acid) (DTNB, Sigma-Aldrich, Taufkirchen, Germany) as a chromogenic reagent. During the reaction, the enzymatic hydrolysis of the substrate produces thiocholine, which reacts with DTNB to form the yellow-colored anion 5-thio-2-nitrobenzoate. The reaction was allowed to proceed for 8 min and was subsequently stopped by adding 10% sodium dodecyl sulfate (SDS). The formation of the reaction product was quantified spectrophotometrically by measuring absorbance at 412 nm. The enzyme concentration was chosen to provide an optimal spectrophotometric signal.

The inhibitory effect of OPs on AChE activity was expressed as the percentage of inhibition (E_i_) according to Equation (1), where E_a0_ represents enzyme activity in the absence of OPs and E_a_ represents enzyme activity measured after exposure to the pesticide solution.

E_i_ = 100% × (E_a0_ − E_a_)/E_a0_ (1)

### 2.4. Blood Sample Preparation

Genotoxicity assessment was conducted using human peripheral blood lymphocytes as a model system. Blood samples were collected from a healthy male donor with informed consent, in accordance with protocols approved by the Institutional Ethics Committee (approval No. 116-5-5/2023-000) and in compliance with health and ethical regulations [37]. Aliquots of whole heparinized blood (0.5 mL) were added to culture tubes containing 4.5 mL of RPMI-1640 medium supplemented with 15% calf serum (Invitrogen-Gibco, Paisley, UK) and 2% phytohemagglutinin. The lymphocyte cultures were treated with prepared carbon materials (final concentrations in blood cultures: 1 µg mL^−1^ and 10 µg mL^−1^). All cultures were set up in triplicate. The untreated cultures served as a control. The data obtained from genotoxicity testing were pooled, and the results are expressed as the mean and standard deviation (SD).

### 2.5. Genotoxicity Testing

Genotoxicity was assessed using the cytokinesis-block micronucleus (CBMN) assay, following the guidelines specified in ISO 10993-3 [38] and Organisation for Economic Cooperation and Development Guideline 487 [39]. For micronuclei (MN) preparation, the CBMN assay of Fenech was performed [40]. Cytochalasin B (Sigma-Aldrich, St. Louis, MO, USA) at a final concentration of 4 µg mL^−1^ was added to each culture 44 h after incubation to inhibit cytokinesis. The lymphocyte cultures were incubated for an additional 28 h. Cells were collected by centrifugation and treated with a hypotonic solution (0.56% KCl + 0.90% NaCl, mixed in equal volumes) at 37 °C. The cell suspension was fixed in methanol/acetic acid (3:1), washed three times with fixative, and dropped onto clean slides. Slides were air-dried and stained with alkaline Giemsa. At least 2000 binucleated cells were scored, and MN were evaluated using an Optech microscope (Munich, Germany) at 400× or 1000× magnification for each sample.

A cytokinesis-block proliferation index (CBPI) was calculated according to a method of Surrales et al. [41] using Equation (2):

CBPI = [MI + 2MII + 3(MIII + MIV)]/N(2)

where MI-MIV represent the number of cells with one to four nuclei, respectively, and N is the number of cells scored. For this assessment, a minimum of 500 cells per slide were evaluated. The proportions of mononucleated, binucleated, trinucleated, and tetranucleated cells in the culture provide a reliable measure of the impact of treatment on cell proliferation and its cytotoxic or cytostatic effects.

### 2.6. Statistics

Statistical analysis of genotoxicity data was performed using one-way ANOVA followed by Tukey’s HSD test for multiple comparisons. Analyses were conducted using Statistica 8 and OriginPro 8.5.1 for Microsoft Windows. Differences were considered statistically significant at *p* < 0.05.

## 3. Results and Discussion

Carbon materials used for adsorption can be broadly categorized into natural, synthetic, and biomass-derived materials. Synthetic carbon materials, such as activated carbons and engineered nanostructures, often provide well-controlled porosity and surface functionality, enabling high adsorption performance. In contrast, biomass-derived carbon materials offer advantages in sustainability, low cost, and waste valorization, though they typically exhibit greater structural heterogeneity. In this context, rabbit litter-derived carbon materials represent a sustainable alternative, where a combination of pore accessibility, surface chemistry, and precursor-derived compositional complexity governs adsorption performance. Naturally occurring carbon materials, such as coal and graphite, have also been investigated for adsorption applications. However, their limited tunability compared to engineered carbon materials often restricts their performance.

### 3.1. Morphology and Elemental Composition

**Figure 1:**
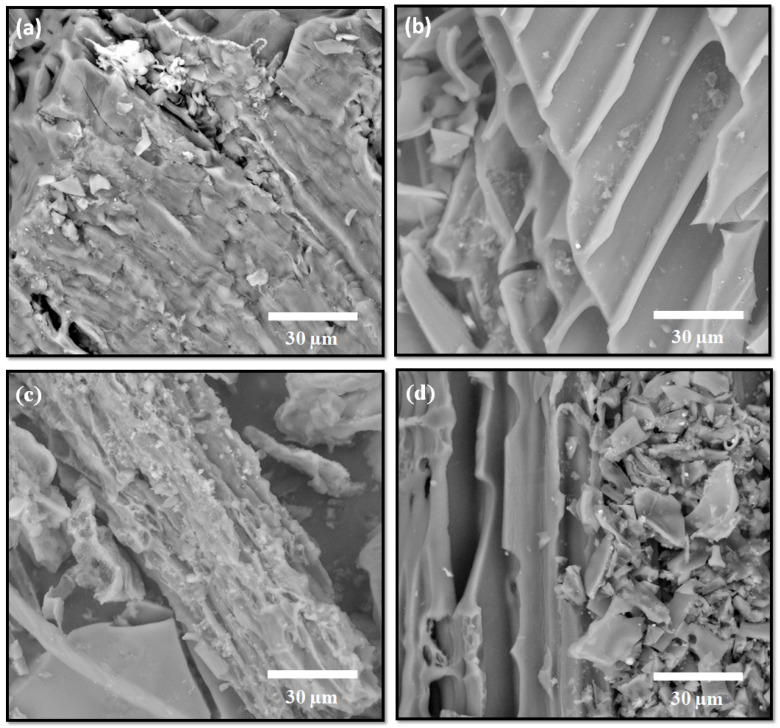
SEM micrographs of materials: (**a**) NRL, (**b**) SRL (U), (**c**) RD, and (**d**) SRL (U,RD) at a magnification of 2500×.

The morphology of the carbon materials derived from different rabbit litter-related precursors was examined by SEM. Representative SEM micrographs of all samples are shown in Figure 1.

As evident from the SEM micrographs, all materials exhibit irregular and heterogeneous morphologies, characteristic of biomass-derived carbons obtained via direct pyrolysis [42]. The carbon obtained from not used rabbit litter (NRL) displays a relatively rough and fragmented surface with numerous surface irregularities. The presence of surface cracks and void-like structures suggests the formation of accessible surface domains, which is consistent with the relatively high specific surface area measured for this sample (results presented below). These morphological features are typical of lignocellulosic biomass-derived carbons and reflect the structural transformation of the original precursor during pyrolysis [17].

In contrast, the carbon derived from spent rabbit litter urine (SRL(U)) exhibits a more compact surface morphology with smoother domains and fewer visible surface cavities. Such a dense structure may reduce the accessibility of internal porosity [43], consistent with the comparatively low textural parameters observed for this material. The rabbit dropping-derived carbon (RD) shows a morphology dominated by granular and agglomerated structures, with smaller surface fissures and a less open pore network compared to NRL. Meanwhile, the carbon obtained from the combined urine and dropping precursor (SRL(U,RD)) exhibits a mixed morphological character, reflecting the heterogeneous composition of the starting material. This sample combines compact regions with localized surface roughness, indicating the coexistence of multiple structural domains.

The elemental composition of the carbonized materials was determined by EDX. It should be noted that EDX analysis provides semi-quantitative information on elemental composition. As shown in Table 1, the NRL sample is characterized by a high carbon content and relatively low oxygen content, indicating a predominantly carbonaceous structure. The SRL(U) sample shows slightly lower carbon content together with increased oxygen and chlorine signals, which may originate from inorganic components present in the urine-derived fraction. In addition, small amounts of Mg, K, and other elements were detected, suggesting the presence of residual mineral components. The RD sample exhibits the highest carbon content among the investigated materials, while detectable amounts of Ca and Cl indicate the presence of mineral phases likely associated with the droppings precursor. Finally, the SRL(U,RD) material shows a more complex elemental composition, with relatively lower carbon content and detectable levels of nitrogen, silicon, chlorine, and other inorganic elements. This compositional heterogeneity reflects the mixed nature of the precursor and may influence the surface chemistry and adsorption behavior of the material. The detected inorganic elements likely originate from the mineral components of the precursor materials, particularly from the urine and droppings fractions, which are known to contain various inorganic salts and residues. Although the NaOH/HCl treatment was primarily applied to remove soluble impurities, it may have influenced the final elemental composition by partially removing or redistributing inorganic species.

### 3.2. FTIR Analysis

**Figure 2:**
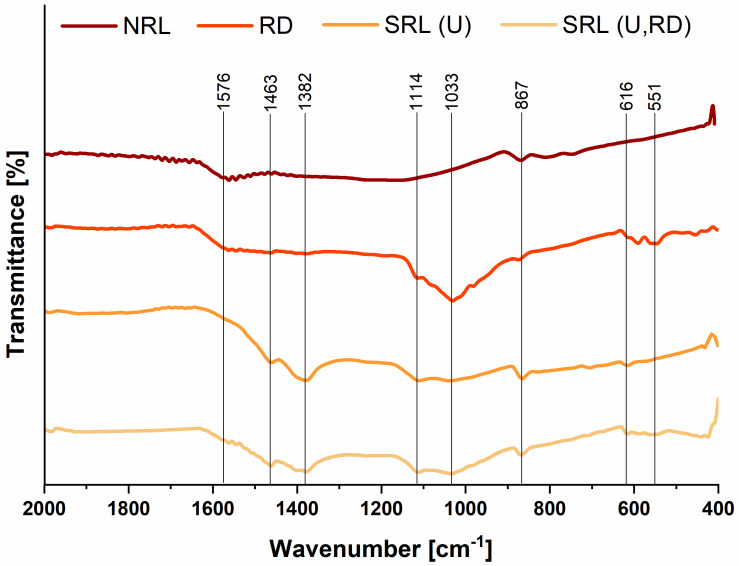
FTIR spectra of carbon materials derived from different rabbit litter precursors (NRL, RD, SRL(U), and SRL(U,RD)) after pyrolysis at 650 °C.

The FTIR spectra of the carbon materials derived from different rabbit litter precursors are presented in Figure 2. The spectra were recorded in the 4000–400 cm^−1^ region, where characteristic vibrations of carbonized biomass structures are typically observed. For clarity, only the 2000–400 cm^−1^ region is shown in Figure 2, as no relevant absorption bands were observed in the 4000–2000 cm^−1^ region. Although the overall spectral profiles are comparable, several differences in band positions and intensities can be observed among the materials, reflecting variations in precursor composition.

A band at approximately 1576 cm^−1^ is observed in the spectra of NRL and RD and is attributed to C=C stretching vibrations of aromatic structures, which commonly arise during the thermal transformation of lignocellulosic biomass [44]. In contrast, bands observed at 1463 and 1382 cm^−1^ are more pronounced in the spectra of SRL(U) and SRL(U,RD) and are generally associated with C-H bending vibrations and residual oxygen-containing functional groups [45]. In the region around 1114 cm^−1^, bands corresponding to C-O stretching vibrations are detected in most samples, except for NRL. These signals may originate from oxygen-containing surface functionalities or contributions from mineral-associated components derived from the precursor materials. A band at 1033 cm^−1^ is clearly observed in the spectrum of RD, which may indicate the presence of C-O vibrations or contributions from inorganic components associated with the droppings-derived precursor [46]. All investigated materials exhibit additional weaker bands detected at 867 cm^−1^ and in the lower wavenumber region (616–551 cm^−1^), which may be related to aromatic C-H out-of-plane bending vibrations and contributions from mineral-associated components originating from the precursor materials [47].

The FTIR results indicate that the investigated carbon materials contain aromatic carbon structures together with residual oxygen-containing functional groups. The differences observed among the spectra reflect variations in precursor composition and the relative contribution of organic and inorganic components, which may influence the surface chemistry and adsorption behavior of the materials toward organophosphate pesticides.

### 3.3. Textural Properties

The nitrogen adsorption–desorption isotherms obtained at 77 K (Figure S1) were employed to assess the textural properties of the synthesized carbon materials. This analysis provided the specific surface area and the BET constant (S_BET_ and C_BET_), the total and micropore volumes (V_tot_ and V_mic_), as well as the median micro- and mesopore diameters (d_mic_ and d_mes_). As shown in Table 2, the textural properties of the carbonized materials varied significantly depending on the precursor composition.

Among the tested samples, NRL exhibited the highest specific surface area (487 m^2^ g^−1^). This, together with an exceptionally high C_BET_ value (4888), a Type I isotherm shape (according to the IUPAC classification [48,49]), and a predominant micropore volume (0.189 cm^3^ g^−1^) relative to the total pore volume (0.205 cm^3^ g^−1^), clearly indicates the highly microporous nature of this material. The exceptionally high C_BET_ value of 4888 suggests very strong adsorbate-adsorbent interactions within the micropores, consistent with Type I isotherm behavior and further supporting the microporous nature of NRL. The slightly positive slope of the adsorption isotherm in the relative pressure range corresponding to mesopores (0.18 < *p*/*p*_0_ < 0.98) suggests the presence of a certain proportion of mesopores (median pore width 16.2 nm), although their contribution is not large. It may pose a challenge for the application of NRL as an adsorbent, given potential diffusion limitations and the possible inaccessibility of the microporous region due to pore blocking by initially adsorbed OP molecules. Indeed, the relatively small mean micropore diameter (0.54 nm) combined with the low mesopore content may render most of the specific surface area of NRL unavailable.

In contrast to the developed microporosity of NRL, the isotherm of the SRL(U) sample exhibits characteristics typical of a Type II isotherm, indicating a non-porous or macroporous material. The limited total pore volume (0.017 cm^3^ g^−1^), low specific surface area (10 m^2^ g^−1^), and small C_BET_ constant (24) further confirm the non-porous nature of SRL(U). These drastic differences likely stem from the chemical composition of the precursors; specifically, the presence of inorganic components in the urine-derived fraction may hinder pore development or cause pore blocking during pyrolysis.

The RD sample exhibited intermediate textural properties, with a specific surface area of 128 m^2^ g^−1^ and a total pore volume of 0.061 cm^3^ g^−1^. A distinguishing feature of its isotherm is a noticeable hysteresis loop across the mesoporous region. The desorption branch runs nearly parallel to the relative pressure axis and closes with the adsorption branch at a *p*/*p*_0_ of approximately 0.42. This shape is characteristic of an H4-type hysteresis loop according to the IUPAC classification, which is typically associated with narrow, slit-like pores in complex micro-mesoporous materials. Given that FTIR analysis revealed the presence of aromatic C=C structures (at ~1576 cm^−1^) in RD, similar to those observed in the lignocellulose-derived NRL, it is plausible that the rigid, anisotropic nature of these aromatic units promotes the formation of slit-shaped interlayer pores during carbonization. It is consistent with the observed H4 hysteresis behavior. Meanwhile, the SRL(U,RD) sample exhibited properties very similar to those of SRL(U), including a low specific surface area (14 m^2^ g^−1^), low total and micropore volumes (0.018 and 0.005 cm^3^ g^−1^, respectively), and a small C_BET_ value (46). It suggests that including the urine fraction in the combined precursor strongly limits porosity development. Overall, it is evident that the precursor composition dictates the final textural characteristics of the carbonized materials, which will fundamentally influence their adsorption behavior towards organophosphate pesticides, as discussed in the subsequent sections.

### 3.4. Adsorption Kinetics

**Figure 3:**
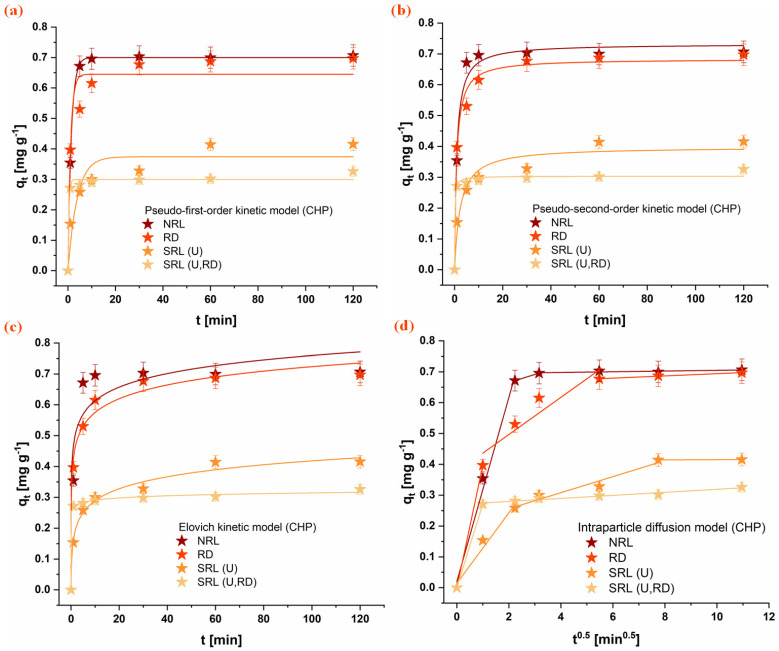
Fitting of kinetic models for CHP adsorption onto the investigated materials: (**a**) PFO, (**b**) PSO, (**c**) Elovich, and (**d**) intraparticle diffusion model. Conditions: 1 mg mL^−1^ adsorbent, 1 × 10^−5^ mol dm^−3^ OP, 25 °C. (NRL—unused rabbit litter; SRL(U)—spent rabbit litter urine; RD—rabbit droppings; SRL(U,RD)—combined precursor).

**Figure 4:**
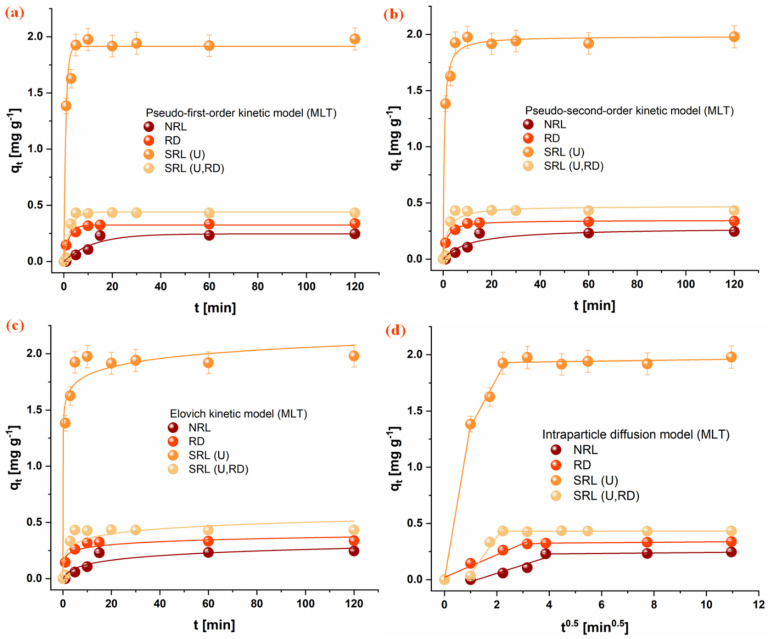
Fitting of kinetic models for MLT adsorption onto the investigated materials: (**a**) PFO, (**b**) PSO, (**c**) Elovich, and (**d**) intraparticle diffusion model. Conditions: 1 mg mL^−1^ adsorbent, 1 × 10^−5^ mol dm^−3^ OP, 25 °C. (NRL—unused rabbit litter; SRL(U)—spent rabbit litter urine; RD—rabbit droppings; SRL(U,RD)—combined precursor).

**Figure 5:**
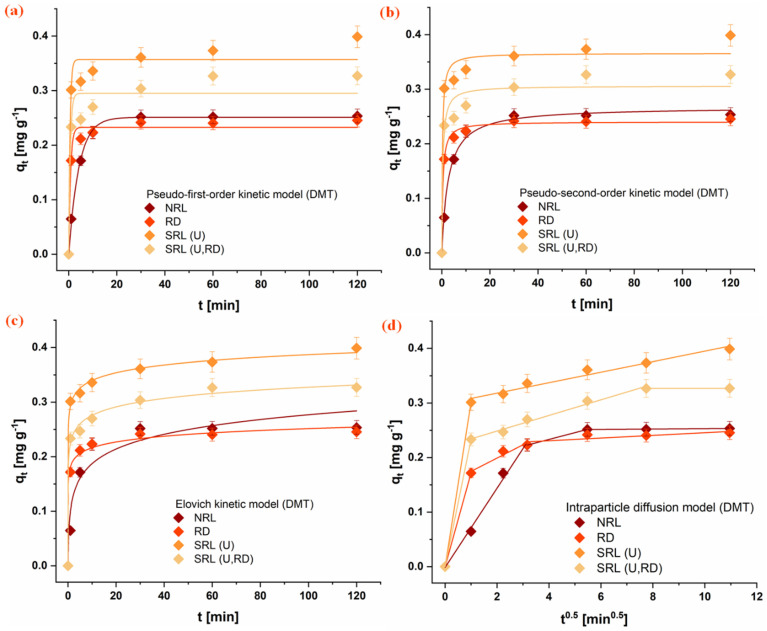
Fitting of kinetic models for DMT adsorption onto the investigated materials: (**a**) PFO, (**b**) PSO, (**c**) Elovich, and (**d**) intraparticle diffusion model. Conditions: 1 mg mL^−1^ adsorbent, 1 × 10^−5^ mol dm^−3^ OP, 25 °C. (NRL—unused rabbit litter; SRL(U)—spent rabbit litter urine; RD—rabbit droppings; SRL(U,RD)—combined precursor).

The adsorption kinetics of the investigated organophosphate pesticides (CHP, MLT, and DMT) onto the rabbit litter-derived carbon materials were evaluated using PFO, PSO, Elovich, and intraparticle diffusion kinetic models. The experimental adsorption profiles are presented in Figure 3, Figure 4 and Figure 5, while the corresponding kinetic parameters are summarized in Table 3. For all investigated systems, a rapid initial adsorption stage was observed during the first phase of the process, indicating rapid interaction between pesticide molecules and available surface sites on the carbon materials. This initial stage was followed by a slower adsorption phase that approached a plateau, suggesting progressive occupation of accessible adsorption domains and the diffusion of pesticide molecules toward internal pores. Based on the experimental adsorption profiles, the majority of adsorption occurred within the first 20–40 min, while equilibrium conditions were reached within 60 min. Therefore, a contact time of 60 min was selected for subsequent adsorption experiments to ensure that equilibrium was achieved for all investigated systems.

Comparison of the kinetic models indicates that both the PFO and PSO models adequately describe the experimental data for most systems. However, the kinetic models should be interpreted primarily as empirical descriptors of adsorption behavior rather than direct evidence of a specific adsorption mechanism [50,51]. The results presented in Table 3 reveal notable differences in adsorption rates depending on both the pesticide and the carbon material. For CHP, the highest adsorption rate was observed for the SRL(U,RD) material, followed by RD and NRL, while the slowest uptake occurred on SRL(U). In contrast, MLT exhibited the fastest adsorption on SRL(U), whereas adsorption on NRL proceeded more slowly. A similar trend was observed for DMT, with SRL(U) and SRL(U,RD) showing the highest adsorption rate constants, while NRL displayed the lowest. These results indicate that adsorption kinetics are not governed solely by surface area, as the material with the highest specific surface area (NRL) did not exhibit the fastest adsorption rates.

The Elovich model further highlights differences in the initial adsorption stage among the materials. In particular, high α values obtained for SRL-derived carbons suggest rapid initial adsorption on energetically heterogeneous surfaces. Although the Elovich parameters should not be interpreted as direct evidence of a specific adsorption mechanism, they indicate that the distribution of surface sites may influence the early stages of pesticide uptake [52]. The intraparticle diffusion model suggests that adsorption occurs through multiple stages. The initial portion of the adsorption process is associated with external surface adsorption or film diffusion, followed by a slower stage attributed to the diffusion of pesticide molecules within the pore structure. This behavior is particularly noticeable for NRL, which exhibits the most developed porous structure among the investigated materials.

An interesting observation from the kinetic analysis is that the material with the highest specific surface area (NRL) did not exhibit the fastest adsorption rates. Instead, faster adsorption was observed for the SRL-derived materials, despite their significantly lower surface areas. It indicates that adsorption kinetics in this system are not solely governed by total surface area but are strongly influenced by pore accessibility. In the predominantly microporous NRL sample, a large fraction of the surface is within narrow pores, limiting mass transfer and slowing adsorption due to diffusion constraints. In contrast, the SRL-derived materials, although possessing low surface areas, provide readily accessible adsorption sites on their external surfaces, enabling faster adsorption.

Differences in adsorption behavior among the investigated pesticides further suggest that molecular structure also plays an important role. The aliphatic organophosphates DMT and MLT exhibit similar adsorption trends across the materials. In contrast, the aromatic CHP shows behavior more closely related to specific surface area, likely due to stronger interactions with graphitic domains. Although adsorption capacities are not strictly proportional to surface area, this may be attributed to partial pore blocking, in which adsorbed molecules in narrow micropores limit access to internal adsorption sites [53].

### 3.5. Adsorption Isotherms

**Figure 6:**
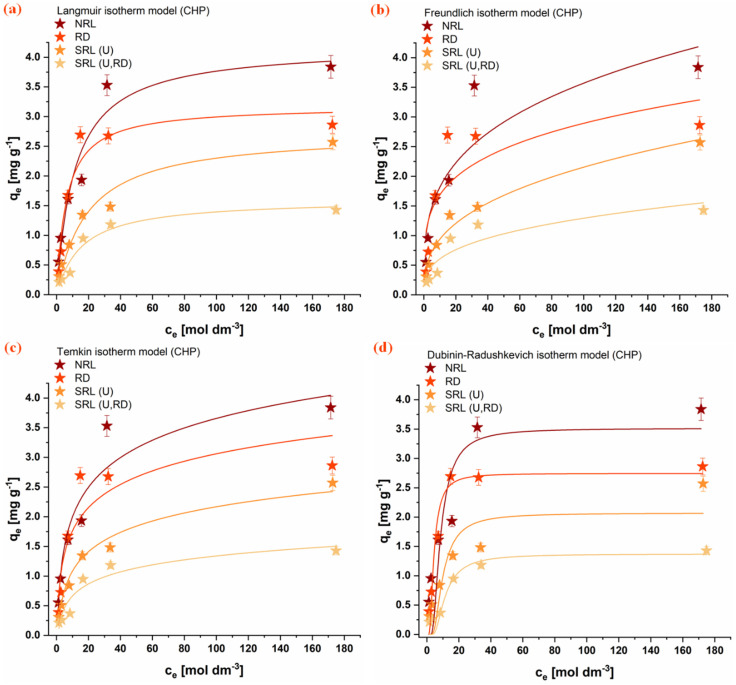
Fitting of isotherm models for CHP adsorption onto the investigated materials: (**a**) Freundlich, (**b**) Langmuir, (**c**) Temkin, and (**d**) Dubinin-Radushkevich. Conditions: 1 mg mL^−1^ adsorbent, 25 °C. (NRL—unused rabbit litter; SRL(U)—spent rabbit litter urine; RD—rabbit droppings; SRL(U,RD)—combined precursor).

**Figure 7:**
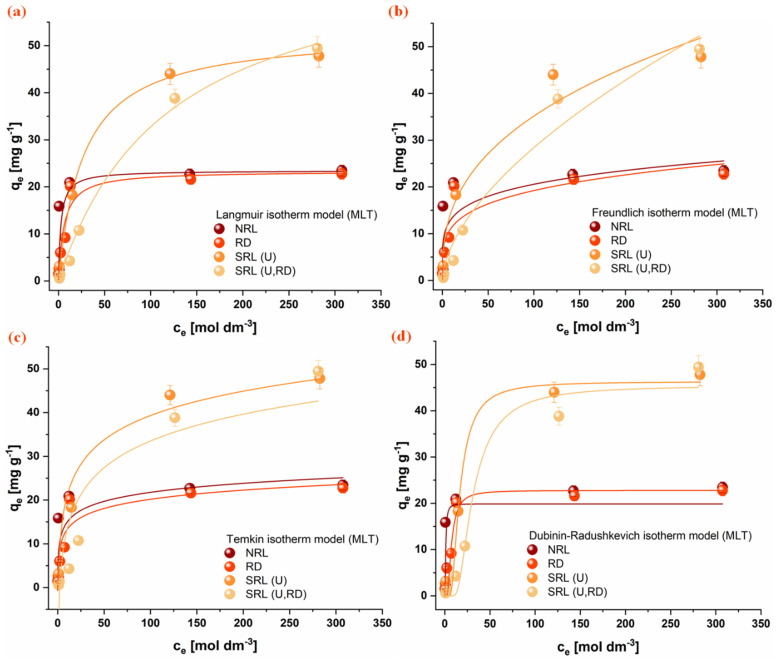
Fitting of isotherm models for MLT adsorption onto the investigated materials: (**a**) Freundlich, (**b**) Langmuir, (**c**) Temkin, and (**d**) Dubinin-Radushkevich. Conditions: 1 mg mL^−1^ adsorbent, 25 °C. (NRL—unused rabbit litter; SRL(U)—spent rabbit litter urine; RD—rabbit droppings; SRL(U,RD)—combined precursor).

**Figure 8:**
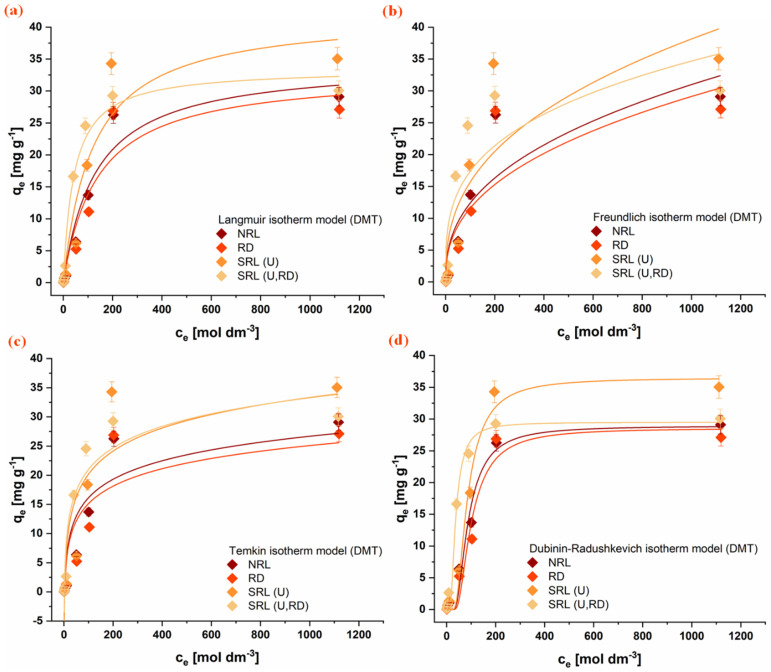
Fitting of isotherm models for DMT adsorption onto the investigated materials: (**a**) Freundlich, (**b**) Langmuir, (**c**) Temkin, and (**d**) Dubinin-Radushkevich. Conditions: 1 mg mL^−1^ adsorbent, 25 °C. (NRL—unused rabbit litter; SRL(U)—spent rabbit litter urine; RD—rabbit droppings; SRL(U,RD)—combined precursor).

The equilibrium adsorption behavior of the investigated organophosphate pesticides (CHP, MLT, and DMT) onto rabbit litter-derived carbon materials was evaluated using several nonlinear isotherm models, including Langmuir, Freundlich, Temkin, and Dubinin-Radushkevich models. The experimental adsorption isotherms together with the corresponding model fits are presented in Figure 6, Figure 7 and Figure 8. The obtained parameters are summarized in Table 4.

Among the tested models, the Langmuir model provided the most consistent fit for all investigated adsorption systems, indicating that it adequately describes the equilibrium adsorption behavior of the studied pesticides on the carbon materials. In several cases, the Freundlich model also showed good agreement with the experimental data, suggesting the presence of heterogeneous adsorption sites on the investigated surfaces. In contrast, the Temkin model generally exhibited poorer agreement, and its parameters were therefore not considered sufficiently reliable for detailed interpretation. The Dubinin-Radushkevich model showed relatively good agreement across most systems, and the adsorption energy values obtained (E, Table 4) indicate that physical interactions dominate the adsorption process [47].

Comparison of the Langmuir adsorption capacities reveals notable differences among the investigated pesticides and materials. For the aromatic pesticide CHP, the adsorption capacity follows the order NRL > RD > SRL(U) > SRL(U,RD), with the highest capacity observed for NRL (4.19 ± 0.07 mg g^−1^). This trend is consistent with the significantly higher specific surface area of this material and suggests that adsorption of this hydrophobic aromatic compound is largely governed by the availability of carbon surface sites and possible π-π interactions with the carbonaceous structure [54,55].

In contrast, the aliphatic OPs MLT and DMT exhibit a different adsorption pattern. For MLT, the highest adsorption capacity was obtained for SRL(U,RD) (71.8 ± 0.1 mg g^−1^), followed by SRL(U) (52.9 ± 0.1 mg g^−1^), despite their considerably lower specific surface areas. A similar tendency is observed for DMT, where SRL(U) shows the highest adsorption capacity (42.1 ± 0.9 mg g^−1^). In contrast, the NRL and RD samples exhibit comparable adsorption capacities for both MLT and DMT, despite their differences in specific surface area, suggesting that the available surface in these materials is not fully accessible. In the case of NRL, this behavior may be attributed to the predominance of micropores and a limited contribution of mesopores, which restricts diffusion of aliphatic molecules and reduces effective utilization of the internal surface [56]. Additionally, partial pore blocking by adsorbed molecules within narrow micropores may further limit access to adsorption sites [57]. These results indicate that adsorption of aliphatic OPs is not solely governed by surface area, but also by the accessibility of adsorption sites and surface chemical characteristics.

Among all investigated systems, the highest overall adsorption capacity was obtained for MLT on the SRL(U,RD) material. The adsorption results suggest that different mechanisms may dominate depending on the pesticide’s structure. While adsorption of the aromatic pesticide CHP appears to be largely governed by surface area and hydrophobic interactions with the carbon framework, adsorption of the more polar and aliphatic pesticides MLT and DMT is likely influenced to a greater extent by specific interactions with oxygen-containing functional groups and heterogeneous surface sites present on the SRL-derived materials.

Carbon materials derived from animal waste precursors have been widely investigated for the removal of various contaminants, including dyes, heavy metals, and certain classes of organic pollutants [58,59,60]. These studies demonstrate that such materials can exhibit favorable adsorption properties due to their inherent mineral content, surface heterogeneity, and tunable porosity. However, investigations focusing specifically on organophosphate pesticides remain relatively limited. In this context, the present study contributes to expanding the application of animal waste-derived carbon materials toward the removal of environmentally relevant organophosphates, for which comparative data remain scarce.

### 3.6. Reduction in Neurotoxicity After Adsorption

The biological relevance of pesticide removal was evaluated using the Ellman assay [36,61] by measuring AChE inhibition before and after adsorption. At an initial concentration of 1 × 10^−5^ mol dm^−3^, the investigated pesticides inhibited AChE activity by 40%, 35%, and 20% for CHP, MLT, and DMT, respectively. After adsorption for 60 min in the presence of the investigated carbon materials (1 mg mL^−1^), a decrease in AChE inhibition was observed for all pesticide-material combinations (Table 5). Although the reduction in inhibition was moderate, the consistent decrease across all systems indicates that adsorption reduces the biologically active fraction of the pesticides in solution. Importantly, no increase in enzyme inhibition was observed after adsorption, indicating that no increase in neurotoxic potential was observed after adsorption under the tested conditions.

Among the investigated systems, the most pronounced reduction in enzyme inhibition was observed for MLT in the presence of SRL(U) and for DMT on all investigated materials, indicating measurable removal of the biologically active pesticide fraction. In contrast, the reduction in inhibition for CHP was more moderate, which may reflect its relatively strong interaction with the enzyme and its higher hydrophobicity compared to the other pesticides. These results demonstrate that adsorption not only reduces the concentration of the investigated OP in solution but also significantly decreases their biological activity, highlighting the potential of the investigated carbon materials to mitigate the neurotoxic effects of environmentally relevant xenobiotics.

### 3.7. Genotoxicity Assessment of Carbon Materials

**Figure 9:**
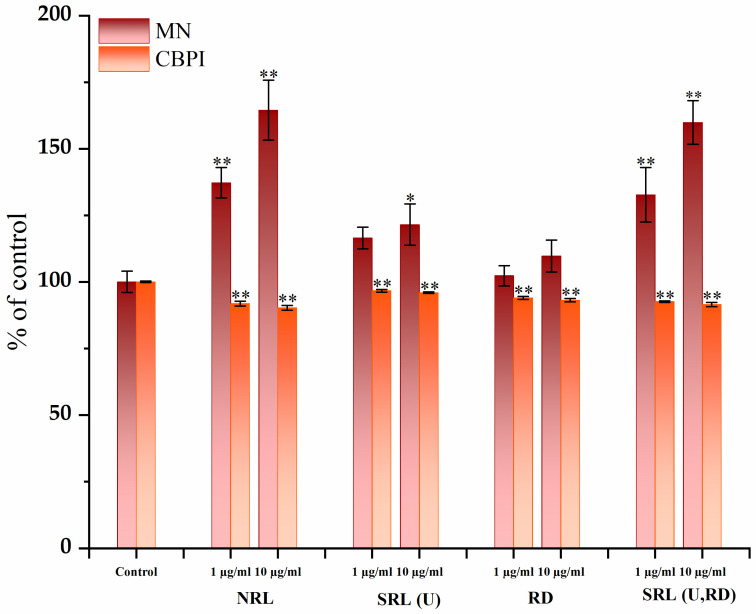
Genotoxicity assessment of investigated carbon materials. Incidence of MN (mean ± SD) and CBPI (mean ± SD), expressed as a percentage of control, set to 100%. Statistical analysis was performed using one-way ANOVA followed by Tukey’s HSD post hoc test. Asterisks indicate statistically significant differences versus control (* *p* < 0.05, ** *p* < 0.001).

The genotoxicity of the tested carbon materials was assessed using the CBMN assay on cultured human peripheral blood lymphocytes. The results demonstrated that carbon materials caused a significant increase (*p* < 0.05) in MN incidence (F(8,18) = 31.37, *p* < 0.001, η^2^ = 0.93) and a decrease (F(8,18) = 70.22, *p* < 0.001, η^2^ = 0.97) in CBPI, in a concentration-dependent manner, indicating both genotoxic and antiproliferative effects. Among the materials tested, RD and SRL (U) induced mild genotoxic effects, while SRL (U,RD) and NRL exhibited a more pronounced genotoxic potential. The obtained results are presented in Table 6 and Figure 9.

The findings suggest that both surface accessibility and surface chemistry, rather than surface area alone, determine the genotoxic potential of tested materials. In other words, the results do not indicate a simple linear relationship between specific surface area and micronuclei incidence. Instead, the data suggest that the increase in micronuclei incidence is likely influenced by both structural and chemical factors. In the highly porous NRL sample, increased biological interaction may be associated with the large accessible interfacial area, whereas in the low-surface-area SRL(U,RD) sample, elevated micronucleus formation may reflect the contribution of chemically heterogeneous mineral- and heteroatom-containing surface domains. These findings indicate that genotoxic effects are determined not solely by porosity, but by the combined influence of textural accessibility and surface compositional complexity. Importantly, these results do not imply that the investigated materials are unsuitable for environmental applications. Rather, they highlight that adsorption efficiency alone may not fully capture the overall environmental performance of waste-derived carbon materials. The observed differences in genotoxic effects among the materials emphasize that both biological impacts and adsorption properties should be assessed during the development of carbon-based adsorbents for environmental remediation.

It should be noted that the genotoxicity assessment was conducted using peripheral blood lymphocytes from a single donor. While this experimental design enabled a controlled evaluation of material-cell interactions and facilitated the detection of treatment-related effects, it does not account for potential inter-individual variability in biological responses and may therefore limit the generalizability of the findings. Nevertheless, the consistent concentration-dependent trends observed across treatments support the interpretation that the effects primarily reflect the intrinsic properties of the tested materials under the applied in vitro conditions. Further studies involving multiple donors are warranted to confirm the broader biological relevance of these findings.

## 4. Conclusions

This study demonstrates that rabbit litter-derived carbon materials can remove OPs from aqueous systems while simultaneously reducing their biological activity. The investigated materials exhibited a broad range of textural properties, with specific surface areas ranging from 10 to 487 m^2^ g^−1^, resulting in markedly different adsorption behaviors. The highest adsorption capacity was observed for malathion on the SRL(U,RD) material (71.8 mg g^−1^), highlighting the importance of precursor composition. The adsorption results reveal that adsorption performance is not governed solely by surface area. Instead, a combination of pore structure, pore accessibility, surface chemistry, and precursor-derived compositional heterogeneity determines the adsorption behavior of the investigated materials. In particular, the aromatic pesticide CHP showed adsorption trends more closely related to surface area and hydrophobic interactions, whereas the aliphatic pesticides MLT and DMT exhibited stronger dependence on surface chemical characteristics and site accessibility. The Ellman assay confirmed that adsorption of the pesticides leads to a measurable decrease in AChE inhibition, with values reduced from 40% to 20% for CHP, from 35% to 11% for MLT, and from 20% to 10% for DMT, demonstrating a reduction in their neurotoxic potential. Importantly, no increase in enzyme inhibition was observed after adsorption, suggesting that contact with the carbon materials does not increase the neurotoxic potential of the pesticides. The genotoxicity assessment further indicates that biological interactions of the materials depend on both textural accessibility and surface compositional complexity. These observations highlight that adsorption efficiency alone does not fully describe the environmental performance of carbon-based adsorbents. Taken together, the results emphasize the importance of integrating physicochemical characterization with biological evaluation when developing waste-derived carbon materials for environmental remediation. Such an approach enables a more comprehensive assessment of both contaminant removal efficiency and potential biological impacts of the materials themselves.

## Figures and Tables

**Table 1:** Elemental composition of carbonized materials determined by EDX analysis. (at.%—atomic percent; ND—under the limit of detection, ranging between 0.1–1 at.%).

Sample	C (at.%)	O (at.%)	Al (at.%)	Cl (at.%)	Ca (at.%)	Mg (at.%)	Si (at.%)	P (at.%)	S (at.%)	Na (at.%)	K (at.%)	N (at.%)
NRL	93.9	4.4	0.7	0.4	0.1	0.2	0.1	0.1	0.1	0.1	ND	ND
SRL (U)	92.9	5.3	ND	1.0	0.1	0.1	ND	ND	0.2	0.2	0.1	ND
RD	98.3	ND	ND	0.5	0.5	ND	0.2	0.2	0.2	ND	0.2	ND
SRL (U,RD)	88.8	4.8	0.3	1.2	0.1	0.2	1.7	0.1	0.2	0.3	0.2	2.1

**Table 2:** Textural properties of carbonized materials (S_BET_—specific surface area; C_BET_—the BET constant; V_tot_—the total volume; V_mic_—micropore volume; *d*_mic_—median micropore diameter; *d*_mes_—median mesopore diameter).

Sample	*S*_BET_ (m^2^ g^−1^)	*C*_BET_	*V*_tot_ (cm^3^ g^−1^)	*V*_mic_ (cm^3^ g^−1^)	*d*_mic_ (nm)	*d*_mes_ (nm)
NRL	487	4888	0.205	0.189	0.54	16.2
SRL (U)	10	24	0.017	0.004	1.21	7.7
RD	128	95	0.061	0.045	0.87	4.5
SRL (U,RD)	14	46	0.018	0.005	1.02	33.6

**Table 3:** Kinetic parameters of OPs adsorption onto 1 mg mL^−1^ materials (q_e_—the quantity of OP adsorbed at equilibrium; k_1_—rate constant for the pseudo-first-order model; k_2_—rate constant for the pseudo-second-order model; α—the initial adsorption rate; β—the desorption constant; k_id_—the adsorption rate constant; C—boundary layer parameter).

		CHP				MLT				DMT			
		NRL	RD	SRL (U)	SRL (U,RD)	NRL	RD	SRL (U)	SRL (U,RD)	NRL	RD	SRL (U)	SRL (U,RD)
pseudo-first-order kinetic model	q_e_ (mg g^−1^)	0.700 ± 0.001	0.645 ± 0.006	0.37 ± 0.02	0.300 ± 0.002	0.247 ± 0.009	0.325 ± 0.003	1.91 ± 0.04	0.441 ± 0.008	0.251 ± 0.001	0.233 ± 0.002	0.357 ± 0.005	0.295 ± 0.008
	k_1_ (min^−1^)	0.698 ± 0.001	0.897 ± 0.004	0.258 ± 0.001	2.37 ± 0.03	0.082 ± 0.008	0.461 ± 0.002	1.17 ± 0.03	0.380 ± 0.009	0.237 ± 0.001	1.33 ± 0.03	1.85 ± 0.05	1.56 ± 0.08
	χ^2^	2.44 × 10^−5^	0.004	0.002	0.002	0.001	0.004	0.009	0.002	3.42 × 10^−5^	0.002	0.008	0.001
	R^2^	0.999	0.943	0.888	0.982	0.914	0.979	0.977	0.928	0.997	0.979	0.955	0.922
pseudo-second-order kinetic model	q_e_ (mg g^−1^)	0.732 ± 0.002	0.683 ± 0.002	0.398 ± 0.006	0.303 ± 0.001	0.28 ± 0.02	0.347 ± 0.001	1.98 ± 0.02	0.47 ± 0.04	0.267 ± 0.001	0.240 ± 0.001	0.366 ± 0.004	0.306 ± 0.006
	k_2_ (mg min^−1^ g^−1^)	1.52 ± 0.01	1.71 ± 0.02	1.06 ± 0.05	24.2 ± 0.2	0.32 ± 0.03	2.09 ± 0.01	1.14 ± 0.02	1.1 ± 0.5	1.39 ± 0.01	9.59 ± 0.02	10.4 ± 0.7	8.08 ± 0.05
	χ^2^	0.001	0.009	0.001	0.002	0.001	8.28 × 10^−5^	0.004	0.004	6.71 × 10^−5^	4.39 × 10^−5^	0.005	0.006
	R^2^	0.986	0.986	0.953	0.987	0.885	0.995	0.9896	0.868	0.994	0.994	0.970	0.951
Elovich kinetic model	α (mg g^−1^ min^−1^)	98.0 ± 0.9	47.6 ± 0.2	0.950 ± 0.002	(2.78 ± 0.01) × 10^9^	0.04 ± 0.01	5.25 ± 0.08	(2.34 ± 0.04) × 10^5^	1.7 ± 0.8	0.464 ± 0.008	(2.26 ± 0.01) × 10^3^	(3.39 ± 0.01) × 10^4^	(4.99 ± 0.01) × 10^2^
	β (g mg^−1^)	15.7 ± 0.9	15.5 ± 0.3	17.8 ± 0.2	98.4 ± 0.1	16 ± 1	26.2 ± 0.9	9.35 ± 0.04	16 ± 8	25.6 ± 0.09	65.7 ± 0.1	48.9 ± 0.1	44.7 ± 0.1
	χ^2^	0.007	0.001	0.003	3.64 × 10^−5^	0.002	0.126	0.017 ± 0.005	0.009	0.008	6.68 × 10^−5^	4.96 × 10^−5^	8.92 × 10^−5^
	R^2^	0.905	0.984	0.986	0.997	0.838	0.924	0.960	0.718	0.922	0.991	0.997	0.993
Intraparticle diffusion kinetic model	C_1_ (mg g^−1^)	0.020 ± 0.002	0	0.014 ± 0.005	3.93 × 10^−17^	0.09 ± 0.01	0.021 ± 0.004	7.85 × 10^−17^	0.281 ± 0.007	0.002 ± 0.001	9.81 × 10^−18^	0	0
	k_id1_ (mg g^−1^ min^−0.5^)	0.300 ± 0.003	0.400	0.114 ± 0.006	0.272	0.07 ± 0.01	0.100 ± 0.005	1.384	0.330 ± 0.007	0.073 ± 0.008	0.172	0.301	0.233
	R^2^	0.983	/	0.943	/	0.83766	0.95858	/	0.93575	0.9909	/	/	/
	C_2_ (mg g^−1^)	0.613	0.38 ± 0.08	0.204 ± 0.005	0.270 ± 0.004	0.22 ± 0.09	0.288	0.933 ± 0.005	0.431 ± 0.008	0.18 ± 0.04	0.150 ± 0.009	0.300 ± 0.006	0.220 ± 0.002
	k_id2_ (mg g^−1^ min^−0.5^)	0.026	0.06 ± 0.02	0.026 ± 0.002	0.005 ± 0.001	0.002 ± 0.001	0.010	0.430 ± 0.005	(1.96 ± 0.09) ×10^−4^	0.03 ± 0.01	0.024 ± 0.009	0.010 ± 0.004	0.014 ± 0.003
	R^2^	/	0.824	0.928	0.946	0.702	/	0.945	0.925	0.869	0.907	0.957	0.979
	C_3_ (mg g^−1^)	0.69 ± 0.01	0.658 ± 0.002	0.411	/	/	0.319 ± 0.001	1.93 ± 0.09	/	0.250	0.22 ± 0.05	/	0.326
	k_id3_ (mg g^−1^ min^−0.5^)	0.001 ± 0.001	0.004 ± 0.001	4.14 × 10^−4^	/	/	0.002 ± 0.001	0.003 ± 0.001	/	3.41 × 10^−4^	0.002 ± 0.001	/	9.36 × 10^−5^
	R^2^	0.895	0.983	/	/	/	0.993	0.914	/	/	0.850	/	/

**Table 4:** Isotherm parameters of OPs adsorption onto 1 mg mL^−1^ materials (K_F_—constant describing adsorption capacity; n—constant describing adsorption intensity; K_L_—the Langmuir constant; q_max_—the theoretical maximum adsorption capacity of the monolayer; b_T_—constant related to the heat of adsorption; K_T_—the equilibrium binding constant; q_DR_—the theoretical saturation capacity; K_DR_—the constant associated with the mean free energy per mole of adsorbent).

		CHP				MLT				DMT			
		NRL	RD	SRL (U)	SRL (U,RD)	NRL	RD	SRL (U)	SRL (U,RD)	NRL	RD	SRL (U)	SRL (U,RD)
Freundlich isotherm model	n	3.4 ± 0.5	4 ± 1	2.77 ± 0.04	3.0 ± 0.8	5 ± 2	4.3 ± 0.9	2.67 ± 0.06	1.69 ± 0.04	2.5 ± 0.7	2.5 ± 0.6	2.6 ± 0.8	3.3 ± 0.7
	K_F_ ((mg g^−1^)(dm^3^ g^−1^)^1/n^)	0.92 ± 0.07	1.0 ± 0.3	0.409 ± 0.005	0.28 ± 0.09	8 ± 3	6.6 ± 0.9	6.26 ± 0.07	1.86 ± 0.04	2.0 ± 0.8	1.8 ± 0.8	2.6 ± 0.8	4.4 ± 0.8
	χ^2^	0.339	0.459	0.023	0.056	39.989	18.773	29.020	16.616	25.628	30.893	53.684	46.638
	R^2^	0.812	0.607	0.966	0.792	0.579	0.781	0.941	0.963	0.823	0.773	0.768	0.746
Langmuir isotherm model	q_max_ (mg g^−1^)	4.19 ± 0.07	3.18 ± 0.08	2.74 ± 0.01	1.61 ± 0.06	23 ± 9	23.4 ± 0.6	52.9 ± 0.1	71.8 ± 0.1	34.6 ± 0.5	33 ± 2	42.1 ± 0.9	33.5 ± 0.3
	K_L_ (dm^3^ mg^−1^)	0.088 ± 0.006	0.161 ± 0.009	0.053 ± 0.005	0.064 ± 0.007	0.3 ± 0.1	0.165 ± 0.009	0.037 ± 0.001	0.008 ± 0.001	0.008 ± 0.002	0.007 ± 0.002	0.008 ± 0.004	0.023 ± 0.002
	χ^2^	0.127	0.087	0.030	0.016	33.139	6.733	0.912	2.636	7.725	13.838	20.425	4.802
	R^2^	0.930	0.925	0.955	0.940	0.651	0.921	0.998	0.994	0.947	0.898	0.912	0.974
Temkin isotherm model	K_t_ (dm^3^ mg^−1^)	1.59 ± 0.09	2.3 ± 0.9	0.997 ± 0.004	0.96 ± 0.02	15 ± 7	8.0 ± 0.6	1.33 ± 0.03	0.44 ± 0.08	0.34 ± 0.08	0.33 ± 0.09	0.34 ± 0.09	0.51 ± 0.01
	b_t_ (J g mol^−1^ mg^−1^)	3440 ± 9	4000 ± 2000	5260 ± 4	8500 ± 100	800 ± 300	800 ± 40	308 ± 3	300 ± 50	500 ± 80	500 ± 200	400 ± 100	460 ± 10
	χ^2^	0.180	0.249	0.022	0.031	34.483	13.120	14.864	80.548	25.803	31.162	48.670	18.584
	R^2^	0.901	0.787	0.967	0.886	0.637	0.847	0.970	0.820	0.822	0.771	0.790	0.899
Dubinin-Radushkevich isotherm model	q_DR_ (mg g^−1^)	4 ± 2	2.75 ± 0.07	2.1 ± 0.9	1.4 ± 0.2	20 ± 10	23 ± 3	46.3 ± 0.1	45.5 ± 0.4	28.9 ± 0.3	28.6 ± 0.5	36.5 ± 0.2	29.5 ± 0.1
	K_DR_ (mol^2^ J^−2^)	(10 ± 5) × 10^−6^	(2.80 ± 0.08) × 10^−6^	(1.3 ± 0.9) × 10^−5^	(1.7 ± 0.1) × 10^−5^	(3 ± 2) × 10^−7^	(7.4 ± 0.4) × 10^−6^	(3.5 ± 0.2) × 10^−5^	(1.22 ± 0.03) × 10^−4^	(9.39 ± 0.03) × 10^−4^	(1.23 ± 0.06) × 10^−3^	(8.81 ± 0.02) × 10^−4^	(1.66 ± 0.02) × 10^−4^
	E (J mol^−1^)	200 ± 100	423 ± 6	200 ± 100	170 ± 10	100 ± 60	260 ± 30	120 ± 10	64.1 ± 0.3	23.1 ± 0.2	20.1 ± 0.5	24 ± 3	54.8 ± 0.1
	χ^2^	0.555	0.085	0.220	0.030	55.219	10.978	5.337	14.348	3.405	5.770	2.893	1.817
	R^2^	0.693	0.928	0.674	0.888	0.418	0.872	0.989	0.968	0.977	0.958	0.988	0.990

**Table 5:** Residual pesticide concentration after adsorption and corresponding AChE inhibition. Removal efficiency was calculated based on the initial pesticide concentration and the residual concentration after adsorption.

OP	Material	Residual OP Concentration (mol dm^−3^)	Removal Efficiency (%)	AChE Inhibition (% of Control)
CHP	before adsorption	1 × 10^−5^	0	40 ± 3
CHP	NRL	8.46 × 10^−6^	15	22 ± 2
CHP	RD	8.24 × 10^−6^	18	20 ± 3
CHP	SRL(U)	8.82 × 10^−6^	12	25 ± 3
CHP	SRL(U,RD)	9.14 × 10^−6^	9	29 ± 2
MLT	before adsorption	1 × 10^−5^	0	35 ± 3
MLT	NRL	9.29 × 10^−6^	7	33 ± 1
MLT	RD	8.99 × 10^−6^	10	31 ± 2
MLT	SRL(U)	4.19 × 10^−6^	58	11 ± 1
MLT	SRL(U,RD)	8.77 × 10^−6^	12	30 ± 4
DMT	before adsorption	1 × 10^−5^	0	20 ± 3
DMT	NRL	8.90 × 10^−6^	11	12 ± 2
DMT	RD	8.95 × 10^−6^	11	12 ± 3
DMT	SRL(U)	8.37 × 10^−6^	16	10 ± 1
DMT	SRL(U,RD)	8.58 × 10^−6^	14	11 ± 1

**Table 6:** Incidence of micronuclei and cytokinesis-block proliferation index in lymphocyte cultures treated with carbon materials. Different superscript letters within the same column indicate statistically significant differences between values according to Tukey’s HSD post hoc test (*p* < 0.05).

Sample		Incidence of Micronuclei MN/1000 BN	Incidence of Micronuclei MN/1000 BN (mean ± S.D.)	CBPI	CBPI (mean ± S.D.)
Control		11.70	11.20 ± 0.45 ^a^	1.88	1.88 ± 0.01 ^a^
		11.06		1.88	
		10.84		1.89	
NRL	1 µg/mL	16.03	15.37 ± 0.64 ^c^	1.71	1.73 ± 0.02 ^de^
		15.33		1.74	
		14.75		1.74	
	10 µg/mL	19.59	18.43 ± 1.26 ^d^	1.68	1.70 ± 0.02 ^d^
		18.61		1.71	
		17.09		1.71	
SRL (U)	1 µg/mL	13.57	13.04 ± 0.45 ^abc^	1.81	1.82 ± 0.01 ^b^
		12.82		1.82	
		12.75		1.83	
	10 µg/mL	14.58	13.61 ± 0.87 ^bc^	1.81	1.81 ± 0.01 ^b^
		13.35		1.81	
		12.90		1.80	
RD	1 µg/mL	11.76	11.46 ± 0.43 ^ab^	1.77	1.77 ± 0.01 ^c^
		11.64		1.76	
		10.97		1.78	
	10 µg/mL	12.99	12.29 ± 0.67 ^ab^	1.74	1.75 ± 0.01 ^ce^
		12.23		1.76	
		11.65		1.76	
SRL (U,RD)	1 µg/mL	15.61	14.87 ± 1.14 ^c^	1.75	1.74 ± 0.01 ^ce^
		15.44		1.74	
		13.55		1.74	
	10 µg/mL	18.81	17.91 ± 0.92 ^d^	1.72	1.72 ± 0.02 ^de^
		17.94		1.71	
		16.97		1.74	

## Data Availability

The original contributions presented in this study are included in the article/Supplementary Materials. Further inquiries can be directed to the corresponding author.

## Supplementary Materials

The following supporting information can be downloaded at: https://www.mdpi.com/article/10.3390/jox16030075/s1, Figure S1: N_2_ adsorption–desorption isotherms of the investigated materials; Figure S2: Original SEM image of NRL; Figure S3: Original SEM image of SRL(U); Figure S4: Original SEM image of RD; Figure S5: Original SEM image of SRL(U,RD).

## References

[1] Saad H., Elfeky S.A., El-Gamel N.E.A., Abo Dena A.S. (2025). Organophosphate pesticides: A review on classification, synthesis, toxicity, remediation and analysis. RSC Adv..

[2] Singh N.K., Sanghvi G., Yadav M., Padhiyar H., Christian J., Singh V. (2023). Fate of pesticides in agricultural runoff treatment systems: Occurrence, impacts and technological progress. Environ. Res..

[3] Ávila-Díaz J.A., González-Márquez L.C., Longoria-Espinoza R.M., Ahumada-Cervantes R., Leyva-Morales J.B., Rodríguez-Gallegos H.B. (2021). Chlorpyrifos and Dimethoate in Water and Sediments of Agricultural Drainage Ditches in Northern Sinaloa, Mexico. Bull. Environ. Contam. Toxicol..

[4] Ishag A.E.S.A., Abdelbagi A.O., Hammad A.M.A., Elsheikh E.A.E., Elsaid O.E., Hur J.-H., Laing M.D. (2016). Biodegradation of Chlorpyrifos, Malathion, and Dimethoate by Three Strains of Bacteria Isolated from Pesticide-Polluted Soils in Sudan. J. Agric. Food Chem..

[5] Ore O.T., Adeola A.O., Bayode A.A., Adedipe D.T., Nomngongo P.N. (2023). Organophosphate pesticide residues in environmental and biological matrices: Occurrence, distribution and potential remedial approaches. Environ. Chem. Ecotoxicol..

[6] Zhou W., Li M., Achal V. (2025). A comprehensive review on environmental and human health impacts of chemical pesticide usage. Emerg. Contam..

[7] Xie Q., Xiao Q., Fan X., Mao H., Gao Y., Zhong C., Shi M., Yu P., Huang J., Deng J. (2026). Neurotoxicity and Immunotoxicity of the Insecticide Isofenphos-methyl in Zebrafish (*Danio rerio*). J. Environ. Sci..

[8] Satyam S., Patra S. (2024). Innovations and challenges in adsorption-based wastewater remediation: A comprehensive review. Heliyon.

[9] Tewari S., Kaur J., Tandon S., Das D.K. (2026). Recent advances in functionalized nanocarbon adsorbents for toxic pollutant removal: Challenges and future perspectives. Discov. Nano.

[10] Sathishkumar R., Prakash R. (2026). A review on activated carbons (AC) for CO_2_ capture applications: Preparation, characterisation and surface modification methods. Fuel.

[11] Katnić Đ., Porobić Katnić S., Lazarević-Pašti T., Milenković M., Terzić T., Marinović-Cincović M., Živojinović D. (2023). Sterilized plum pomace biochar as a low-cost effective sorbent of environmental contaminants. J. Water Process Eng..

[12] Milankovic V., Terzić T., Brkovic S., Potkonjak N., Unterweger C., Bajuk-Bogdanović D., Pasti I., Lazarević-Pašti T. (2024). Spent coffee grounds-derived carbon material as an effective adsorbent for removing multiple contaminants from wastewater: A comprehensive kinetic, isotherm, and thermodynamic study. J. Water Process Eng..

[13] Milankovic V., Terzić T., Brkovic S., Potkonjak N., Unterweger C., Pasti I., Lazarević-Pašti T. (2024). The adsorption of chlorpyrifos and malathion under environmentally relevant conditions using biowaste carbon materials. J. Hazard. Mater..

[14] Molla A., Khatun N., Kim D. (2026). Biomass-derived carbon materials as adsorbent materials for CO_2_, organic dyes, antibiotics, and heavy metals-a review. Biomass Futures.

[15] Mohamad Nor N., Lau L.C., Lee K.T., Mohamed A.R. (2013). Synthesis of activated carbon from lignocellulosic biomass and its applications in air pollution control—A review. J. Environ. Chem. Eng..

[16] Terzić T., Milankovic V., Potkonjak N., Unterweger C., Pasti I., Lazarević-Pašti T. (2025). Valorization of viscose textile waste for the adsorptive removal of organophosphate pesticides from water. J. Water Process Eng..

[17] Terzić T., Milankovic V., Unterweger C., Fuerst C., Breitenbach S., Pasti I., Lazarević-Pašti T. (2024). Highly Porous Cellulose-Based Carbon Fibers as Effective Adsorbents for Chlorpyrifos Removal: Insights and Applications. C.

[18] Zieliński B., Miądlicki P., Przepiórski J. (2022). Development of activated carbon for removal of pesticides from water: Case study. Sci. Rep..

[19] Li W.-K., Shi Y.-P. (2024). Recent advances of carbon materials on pesticides removal and extraction based determination from polluted water. TrAC Trends Anal. Chem..

[20] Uddin S. (2021). Removal of Pesticides Using Carbon-Based Nanocomposite Materials.

[21] Gupta S., Ronen Z. (2025). Treatment of Xenobiotic Cyclic Nitramine Explosives in Wastewater. J. Xenobiot..

[22] Štefanac T., Grgas D., Landeka Dragičević T. (2021). Xenobiotics-Division and Methods of Detection: A Review. J. Xenobiot..

[23] Subbulakshmi G., Debbarma A., Sinha A., Panda S. (2021). Bio remediation of xenobiotic compound: Reclamation approach for environmental sustainability—A review. Mater. Today Proc..

[24] Lionetto M.G., Caricato R., Calisi A., Giordano M.E., Schettino T. (2013). Acetylcholinesterase as a biomarker in environmental and occupational medicine: New insights and future perspectives. BioMed Res. Int..

[25] Chen Y., Yang Z., Nian B., Yu C., Maimaiti D., Chai M., Yang X., Zang X., Xu D. (2024). Mechanisms of Neurotoxicity of Organophosphate Pesticides and Their Relation to Neurological Disorders. Neuropsychiatr. Dis. Treat..

[26] Bhattacharya K., Mukherjee S.P., Gallud A., Burkert S.C., Bistarelli S., Bellucci S., Bottini M., Star A., Fadeel B. (2016). Biological interactions of carbon-based nanomaterials: From coronation to degradation. Nanomed. Nanotechnol. Biol. Med..

[27] Awasthi S., Srivastava A., Kumar D., Pandey S.K., Mubarak N.M., Dehghani M.H., Ansari K. (2024). An insight into the toxicological impacts of carbon nanotubes (CNTs) on human health: A review. Environ. Adv..

[28] Lindner K., Ströbele M., Schlick S., Webering S., Jenckel A., Kopf J., Danov O., Sewald K., Buj C., Creutzenberg O. (2017). Biological effects of carbon black nanoparticles are changed by surface coating with polycyclic aromatic hydrocarbons. Part. Fibre Toxicol..

[29] Koike E., Kobayashi T. (2006). Chemical and biological oxidative effects of carbon black nanoparticles. Chemosphere.

[30] Sigmund G., Jiang C., Hofmann T., Chen W. (2018). Environmental transformation of natural and engineered carbon nanoparticles and implications for the fate of organic contaminants. Environ. Sci. Nano.

[31] Rouquerol J., Rouquerol F., Sing K. (2013). Adsorption by Powders and Porous Solids: Principles, Methodology and Applications.

[32] Rouquerol J., Llewellyn P., Rouquerol F. (2007). Is the BET Equation Applicable to Microporous Adsorbents?. Stud. Surf. Sci. Catal..

[33] Rouquerol J., Llewellyn P., Rouquerol F. (2007). Characterization of Porous Solids VII.

[34] HorvÁTh G., Kawazoe K. (1983). Method for Calculation of Effective Pore Size Distribution in Molecular Sieve Carbon. J. Chem. Eng. Jpn..

[35] Barrett E.P., Joyner L.G., Halenda P.P. (1951). The Determination of Pore Volume and Area Distributions in Porous Substances. I. Computations from Nitrogen Isotherms. J. Am. Chem. Soc..

[36] Ellman G.L., Courtney K.D., Andres V., Feather-Stone R.M. (1961). A new and rapid colorimetric determination of acetylcholinesterase activity. Biochem. Pharmacol..

[37] Law on Health Care, Official Gazette of the Republic of Serbia, No. 25/2019-40. https://pravno-informacioni-sistem.rs/eli/rep/sgrs/skupstina/zakon/2019/25/2/reg.

[38] (2014). Biological Evaluation of Medical Devices—Part 3: Tests for Genotoxicity, Carcinogenicity and Reproductive Toxicity.

[39] OECD (2023). Test No. 487: In Vitro Mammalian Cell Micronucleus Test.

[40] Fenech M. (1993). The cytokinesis-block micronucleus technique: A detailed description of the method and its application to genotoxicity studies in human populations. Mutat. Res..

[41] Surrallés J., Xamena N., Creus A., Catalán J., Norppa H., Marcos R. (1995). Induction of micronuclei by five pyrethroid insecticides in whole-blood and isolated human lymphocyte cultures. Mutat. Res./Genet. Toxicol..

[42] Yuan S.-J., Wang J.-J., Dong B., Dai X.-H. (2023). Biomass-Derived Carbonaceous Materials with Graphene/Graphene-Like Structures: Definition, Classification, and Environmental Applications. Environ. Sci. Technol..

[43] Bukka V.V.R., Sarin P. (2024). Effects of Particle Size Reduction on the Pore Structure and Accessibility in Natural Porous Materials. Energy Fuels.

[44] Al Lafi A.G., Khuder A. (2025). Removal of Cr(VI) from aqueous solutions by activated carbon and its composite with P_2_W_17_O_61_: A spectroscopic study to reveal adsorption mechanism. Heliyon.

[45] Milanković M., Mravik Ž., Vasiljević B., Bajuk-Bogdanović D., Uskoković-Marković S., Jovanovic Z. (2026). Chemical Titrations and Temperature-Programmed Desorption Study of the Surface Chemistry of Graphene Oxide and 12-Tungstophosphoric Acid Nanocomposite. Processes.

[46] Subba Reddy Y., Rotte N.K., Sudhakar B.K., Ramakrishna Chand N., Naik R.J., Mandal S., Ravi Chandra M. (2024). Biomass-derived sustainable mesoporous activated carbon as an efficient and recyclable adsorbent for the adsorption of hazardous dyes. Hybrid Adv..

[47] Milanković V., Tasić T., Brković S., Potkonjak N., Unterweger C., Pašti I.A., Lazarević-Pašti T. (2024). Transforming Food Biowaste into Selective and Reusable Adsorbents for Pesticide Removal from Water. Materials.

[48] Thommes M., Kaneko K., Neimark A.V., Olivier J.P., Rodriguez-Reinoso F., Rouquerol J., Sing K.S.W. (2015). Physisorption of gases, with special reference to the evaluation of surface area and pore size distribution (IUPAC Technical Report). Pure Appl. Chem..

[49] Rahman M., Shafiullah A.Z., Pal A., Islam M.A., Jahan I., Saha B. (2021). Study on Optimum IUPAC Adsorption Isotherm Models Employing Sensitivity of Parameters for Rigorous Adsorption System Performance Evaluation. Energies.

[50] Tran H.N., You S.-J., Hosseini-Bandegharaei A., Chao H.-P. (2017). Mistakes and inconsistencies regarding adsorption of contaminants from aqueous solutions: A critical review. Water Res..

[51] Wang J., Guo X. (2020). Adsorption kinetic models: Physical meanings, applications, and solving methods. J. Hazard. Mater..

[52] Ali I., Burakova I., Galunin E., Burakov A., Mkrtchyan E., Melezhik A., Kurnosov D., Tkachev A., Grachev V. (2019). High-Speed and High-Capacity Removal of Methyl Orange and Malachite Green in Water Using Newly Developed Mesoporous Carbon: Kinetic and Isotherm Studies. ACS Omega.

[53] Choma J., Szczęśniak B., Kapusta A., Jaroniec M. (2024). A Concise Review on Porous Adsorbents for Benzene and Other Volatile Organic Compounds. Molecules.

[54] Mogashane T.M., Motlatle M.A., Mkhohlakali A.C., Mokoena L.V., Tshilongo J. (2026). Biochar-Based Adsorption of Polycyclic Aromatic Hydrocarbons in Contaminated Soils: Advances, Mechanisms, and Bibliometric Analysis. Results Eng..

[55] Yang K., Jiang Y., Yang J., Lin D. (2018). Correlations and adsorption mechanisms of aromatic compounds on biochars produced from various biomass at 700 °C. Environ. Pollut..

[56] Dziejarski B., Serafin J., Hernández-Barreto D.F., Naumovska E., Sreńscek-Nazzal J., Klomkliang N., Tam E., Krzyżyńska R., Andersson K., Knutsson P. (2025). Tailoring highly surface and microporous activated carbons (ACs) from biomass via KOH, K_2_C_2_O_4_ and KOH/K_2_C_2_O_4_ activation for efficient CO_2_ capture and CO_2_/N_2_ selectivity: Characterization, experimental and molecular simulation insights. Chem. Eng. J..

[57] Bhatia S.K., Liu F., Arvind G. (2000). Effect of Pore Blockage on Adsorption Isotherms and Dynamics: Anomalous Adsorption of Iodine on Activated Carbon. Langmuir.

[58] Hong J., Kim H., Chae C., Kim D.H., Lee S.O., Kim I. (2025). Sulfamethazine adsorption using livestock manure-derived biochar: Significance of oxygen concentration during biochar generation. Biomass Bioenergy.

[59] Lee S., Kim M., Park G., Jung S., Kwon E. (2025). Animal manure biochar for the removal of hazardous pollutants from wastewater. Biochar X.

[60] Hadroug S., Khiari B., Jellali S., El-Bassi L., Al-Wardy M., Hamdi W., Jeguirim M. (2025). Animal manure derived biochars synthesis, characterization and use for wastewater treatment and in agriculture: A recent review. Sci. Total Environ..

[61] Kumar R., George L., Jun Z., Mukherji S. (2022). Photocatalytic activity of graphene oxide-TiO_2_ nanocomposite on dichlorvos and malathion and assessment of toxicity changes due to photodegradation. Chemosphere.

