# Supplementary material for: Rabbit Litter-Derived Carbon Materials for Organophosphate Pesticide Mitigation: Adsorption Performance, Neurotoxicity Reduction, and Genotoxicity Assessment
Source: J Xenobiot. 2026 Apr 29;16(3):75. doi: 10.3390/jox16030075 (PMC13214656; doi:10.3390/jox16030075)
Supplement: Supplementary file 1 [file jox-16-00075-s001.zip › jox-4274866-supplementary.pdf]

Journal of Xenobiotics

MDPI

Article

Rabbit Litter-Derived Carbon Materials for Organophosphate Pesticide Mitigation: Adsorption Performance, Neurotoxicity Reduction, and Genotoxicity Assessment

Tamara Lazarević-Pašti, Tamara Terzić, Andreja Leskovac, Sandra Petrović, Vedran Milanković, Nevena Radivojević, Jugoslav Krstić, Katarina Kokanov Stanković, Ana Jocic, Snežana Brković and Igor Pašti

Adsorption experiments

The adsorption efficiency was calculated as:

$$\text{Uptake} = 100\% \times (C_0 - C_{eq})/C_0$$

, where C_0 is the starting concentration of OPs and C_{eq} is the equilibrium concentration of OPs.

To study the adsorption kinetics of OPs onto the materials, nonlinear kinetic models, including pseudo-first-order, pseudo-second-order, Elovich, and the intraparticle diffusion model, were employed. Additionally, adsorption isotherms were analyzed using nonlinear models, including the Freundlich, Langmuir, Temkin, and Dubinin-Radushkevich models.

The following equations describe the adsorption kinetics:

Pseudo-first-order model $$q_t = q_e(1 - e^{-k_1 t})$$

Pseudo-second-order model $$q_t = \frac{q_e^2 k_2 t}{1 + q_e k_2 t}$$

Elovich kinetic model $$q_t = \frac{1}{\beta}(1 + \alpha\beta t)$$

Intraparticle diffusion model $$q_t = k_{id} t^{0.5} + C$$

In these equations, q_t represents the quantity of adsorbate adsorbed at time t (mg g^{-1}), and q_e represents the quantity adsorbed at equilibrium (mg g^{-1}). The constants k_1 (min^{-1}) and k_2 (g mg^{-1} min^{-1}) are the rate constants for the pseudo-first-order and pseudo-second-order models, respectively. The Elovich model parameters include α, the initial adsorption rate (mg g^{-1} min^{-1}), and β, the desorption constant (g mg^{-1}). The intraparticle diffusion model uses k_{id} as the adsorption rate constant (mg g^{-1} $min^{-0.5}$) and C as a boundary layer parameter (mg g^{-1}).

These equations describe the adsorption isotherms:

Freundlich model $$q_e = K_F C_e^{\frac{1}{n}}$$

Langmuir model $$q_e = \frac{q_{max} K_L C_e}{1 + K_L C_e}$$

Temkin model $$q_e = \frac{RT}{b_T} ln K_T C_e$$

Dubinin-Radushkevic model $$q_e = q_{DR} e^{-K_{DR} \varepsilon^2}$$

In these equations, q_e represents the quantity adsorbed at equilibrium (mg g^{-1}), while C_e represents the equilibrium adsorbate concentration (mg dm^{-3}). In the Freundlich isotherm model, K_F ((dm^3 mg^{-1})$^{1/n}$) and n are constants describing adsorption capacity and intensity, respectively. The Langmuir model uses K_L (dm^3 mg^{-1}) as the Langmuir constant and q_{max} (mg g^{-1}) as the theoretical maximum adsorption capacity of the monolayer. For the Temkin isotherm, b_T (J g mol^{-1} mg^{-1}) and K_T (dm^3 mg^{-1}) are constants related to the heat of adsorption and the equilibrium binding constant, respectively. The Dubinin-Raduskevic isotherm defines q_{DR} (mg g^{-1}) as the theoretical saturation capacity and K_{DR} (mol^2 J^{-2}) as the constant associated with the mean free energy per mole of adsorbent ($\varepsilon = RT \times \ln(1 + 1/C_e)$).

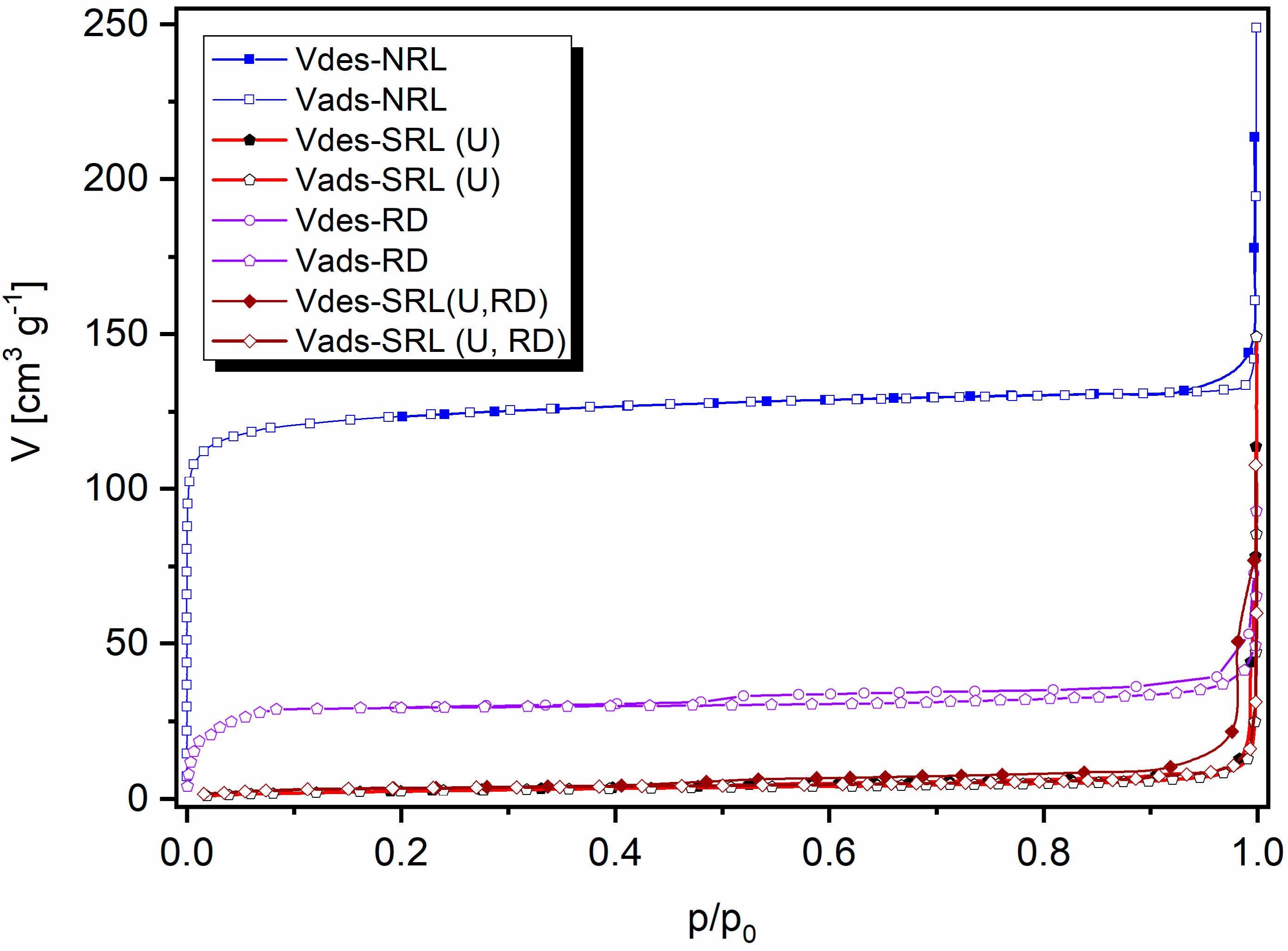

Figure S1. N_2 adsorption-desorption isotherms of investigated materials

Figure S2: Original SEM image of NRL

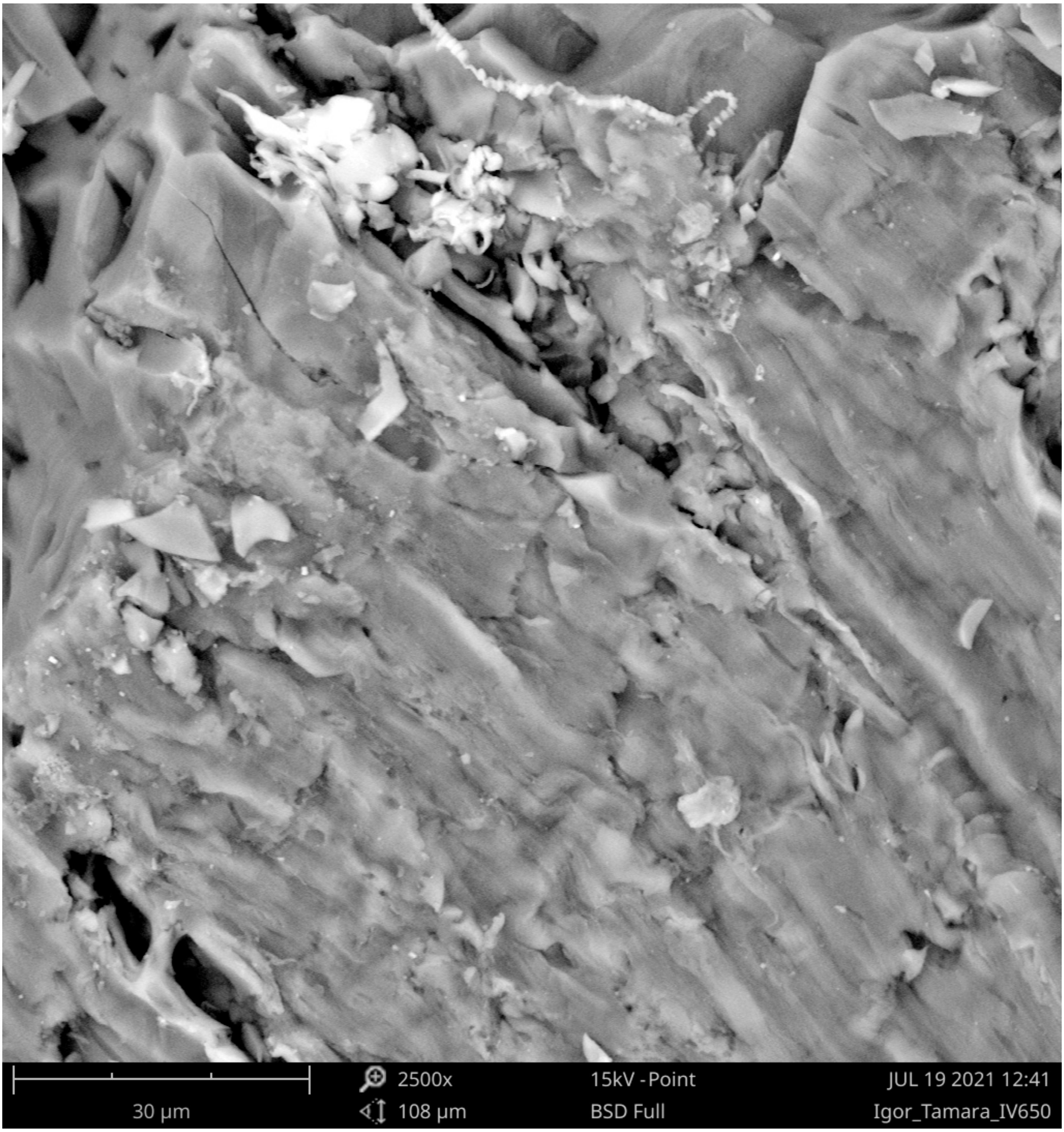

Figure S3: Original SEM image of SRL(U)

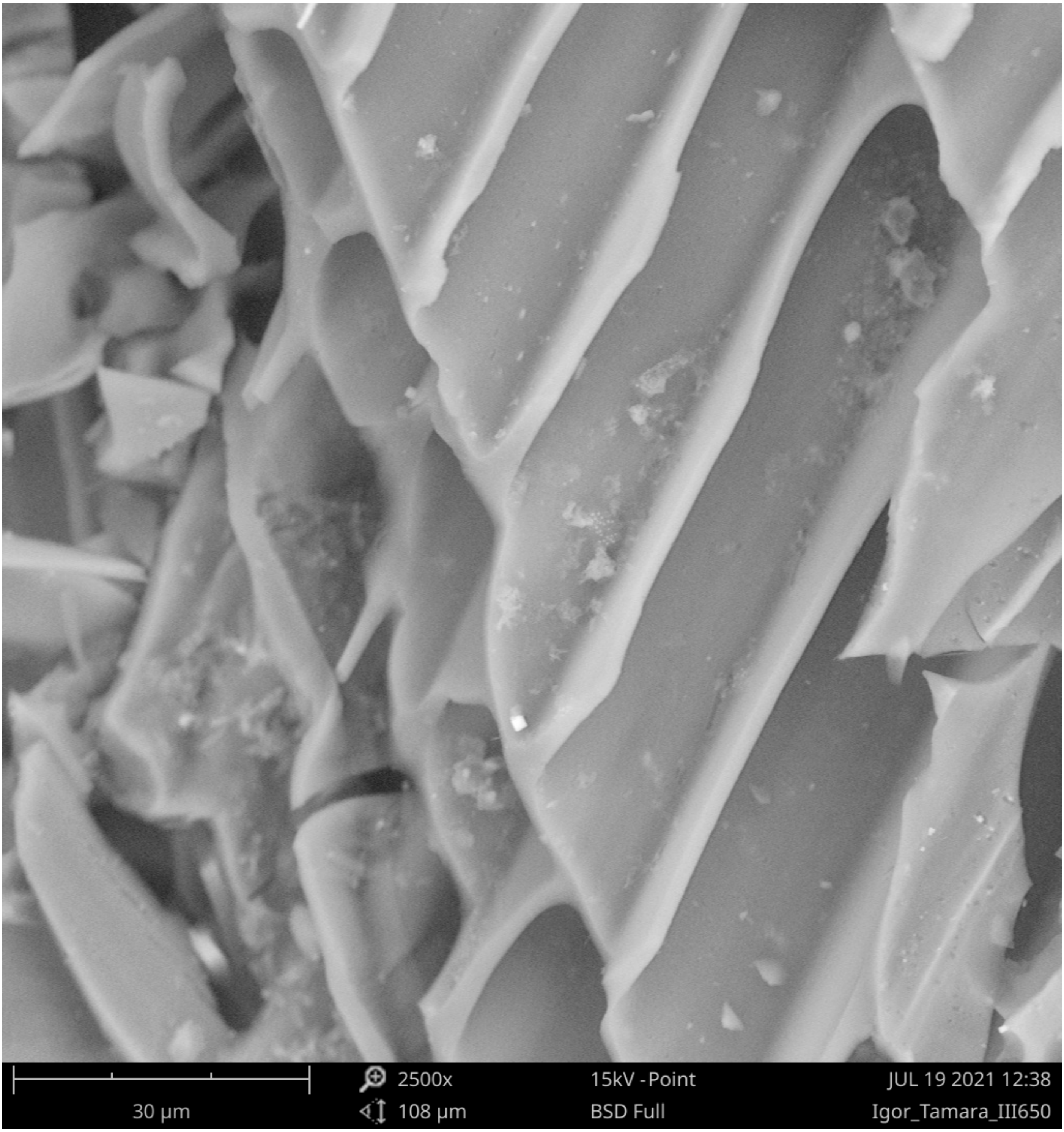

Figure S4: Original SEM image of RD

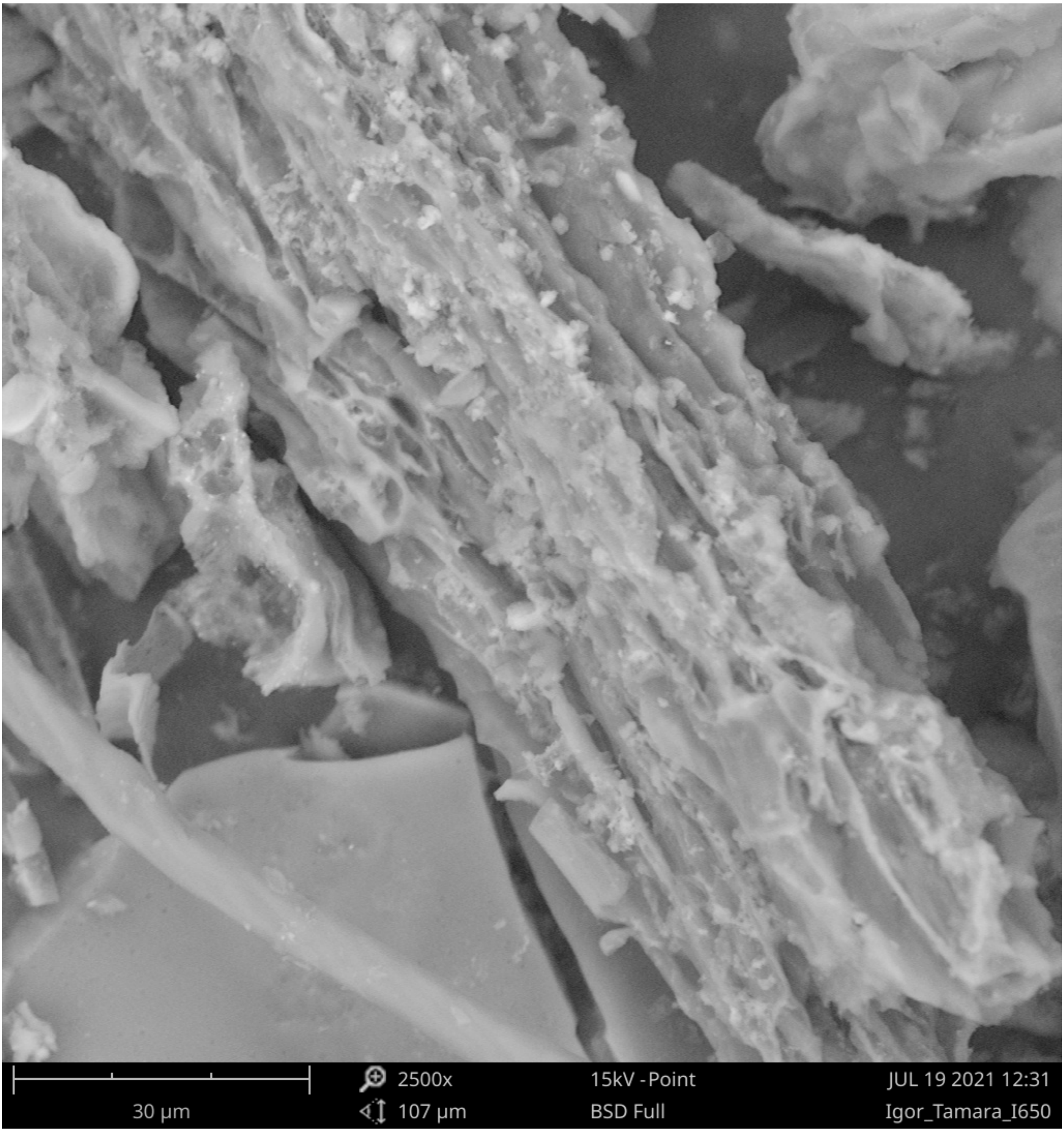

Figure S5: Original SEM image of SRL(U,RD)

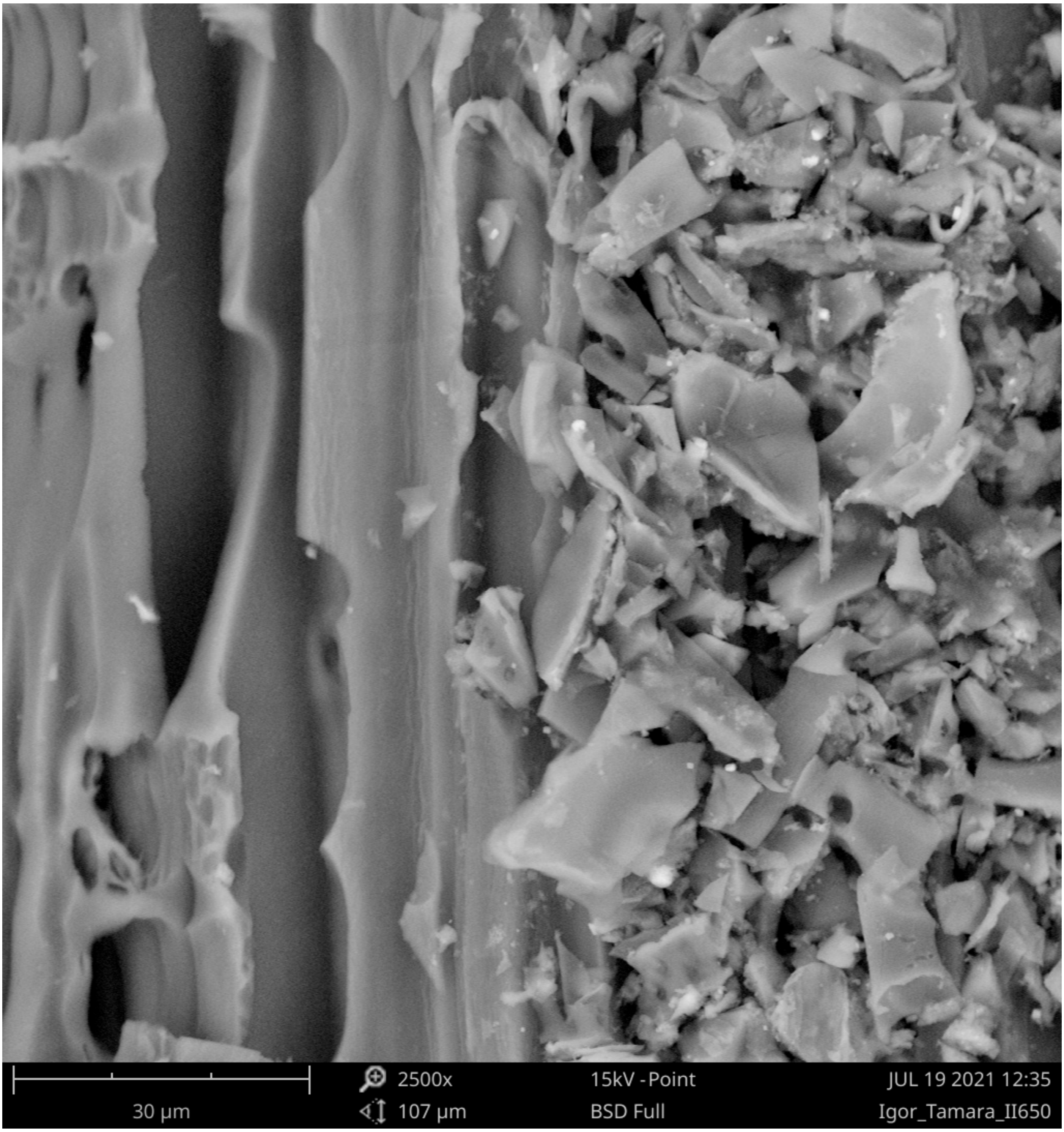

Disclaimer/Publisher's Note: The statements, opinions and data contained in all publications are solely those of the individual author(s) and contributor(s) and not of MDPI and/or the editor(s). MDPI and/or the editor(s) disclaim responsibility for any injury to people or property resulting from any ideas, methods, instructions or products referred to in the content.

https://doi.org/10.3390/jox16030075
